# Changing age profile and incidence of injecting drug use initiation among people in Australia who inject drugs: evidence from two national repeated cross-sectional studies

**DOI:** 10.1016/j.lanwpc.2025.101548

**Published:** 2025-04-17

**Authors:** Olivia Price, Lisa Maher, Amy Peacock, Farzaneh Zolala, Louisa Degenhardt, Paul Dietze, Sarah Larney, Seraina Agramunt, Samantha Colledge-Frisby, Bradley Mathers, Dan Lewer, Rachel Sutherland

**Affiliations:** a National Drug and Alcohol Research Centre, UNSW Sydney, Sydney, Australia; b Kirby Institute, UNSW Sydney, Sydney, Australia; c Burnet Institute, Melbourne, Australia; d School of Psychological Sciences, University of Tasmania, Hobart, Australia; e Public Health Intelligence, Queensland Department of Health, Herston, Australia; f National Drug Research Institute, Curtin University, Melbourne, Australia; g Centre de Recherche du Centre Hospitalier de l’Université de Montréal, Montréal, Canada; h Department of Family Medicine and Emergency Medicine, Faculty of Medicine, University of Montréal, Montréal, Canada; i National Drug Research Institute, Curtin University, Perth, Australia; j Kurongkurl Katitjin, Edith Cowan University, Perth, Australia; k Bradford Institute for Health Research, Bradford, UK; l Department of Epidemiology and Public Health, University College London, London, UK

**Keywords:** Ageing, Demography, Epidemiology, Injecting drug use, Modelling, Australia

## Abstract

**Background:**

The age of people who inject drugs appears to be increasing in some high-income countries. We aimed to explore trends in the age of people injecting and recently starting to inject drugs in Australia, and to model incidence of initiation over time.

**Methods:**

We obtained data from the Illicit Drug Reporting System (IDRS) and the Australian Needle Syringe Program Survey (ANSPS), which comprise annual cross-sectional surveys with people who inject drugs (2000–2019). Outcome measures were current age, age of initiation, and time since initiation (both surveys), and drug first injected (IDRS only). We estimated time trends in age using quantile regression. We used the relative number of people initiating injecting drug use each year and existing population size estimates to model incidence of initiation.

**Findings:**

In total, 58,465 interviews with people who reported injecting drugs in the past month (33.7% women) were included. In both surveys, the median age increased over the study period (IDRS: 28–43 years; ANSPS 28–44 years). The median time since initiation also increased over the study period (IDRS: 8–22.5 years; ANSPS: 9–24 years), and the median age of initiation increased with calendar year of initiation (IDRS: 18–34 years; ANSPS: 20–30 years). Women tended to be younger and commence injecting drug use at a younger age than men. Modelling suggested that similar numbers of people injected drugs for the first time each year from 1980 (national estimates of 15,570 and 17,020 new initiates using IDRS and ANSPS data, respectively) to 1996 (IDRS: 16,360; ANSPS: 22,300 new initiates), followed by a decisive decline in incidence until 2012 (IDRS: 1110; ANSPS: 1830), whereafter it has fluctuated but remained low. Meth/amphetamine was consistently the predominant drug injected at initiation among IDRS participants, although there were peaks in heroin as the drug injected at initiation in the early 1980s and mid-1990s.

**Interpretation:**

In 2019, most people who were injecting drugs in Australia were part of a cohort that began injecting in the 1980s or 1990s. Consequently, the population was older and had been injecting drugs for longer compared to those injecting drugs in 2000. This has implications for health service delivery to people who inject drugs, with increasing age likely to be accompanied by a rise in chronic health conditions and an increase in injecting duration potentially resulting in higher incidence of injecting-related injuries and diseases.

**Funding:**

10.13039/501100003921Australian Government Department of Health and Aged Care.

**Research in context**

**Evidence before this study**

We conducted a PubMed search using terms relating to injecting drug use (any field), ageing or injecting initiation (any field), and epidemiological or population studies (any field) on 11 February 2025. The search was restricted to studies published in the last five years and yielded 407 results. We also reviewed studies included in a scoping review of ageing and older people who use illicit drugs published in 2022 by Zolopa and colleagues. We identified 18 relevant studies. Most (n = 8) used population household survey data to describe the increasing age of people using or injecting drugs, or to project the proportion of older adults (aged ≥50 years) who use illicit or non-prescribed drugs, have substance use disorder, or require drug treatment. Although these studies describe population-level trends in age, household survey data are typically not representative of people who use or inject illicit drugs. Other studies (n = 7) used prospective cohort data to describe patterns in injecting drug use duration and cessation (‘injecting careers’). However, this study design does not allow inference of population-level trends. A literature review of factors associated with age at first injection did not report any studies that assessed the role of calendar time. We found one study which demonstrated that repeat cross-sectional surveys with people who inject drugs can be used to describe population-level patterns in age, age of injecting drug use initiation, and duration since initiation. This study attributed the increasing age of people who inject drugs in the UK to a cohort effect, whereby many people began injecting drugs in the mid-1990s and continue to inject drugs today.

**Added value of this study**

Using twenty years (2000–2019) of data from two Australian national surveys with people who inject drugs (n = 58,465), we described trends in current age, age of injecting drug use initiation, and time since initiation. We also used information on the relative number of people initiating injecting drug use each year to model the incidence of injecting initiation. These analyses show that the age of people currently injecting drugs in Australia increased from the late 20s in 2000 to the early 40s in 2019, while the typical time since initiation increased from approximately 8 years to over 20 years. We demonstrated that incidence of injecting drug use initiation was stable or increasing through the 1980s and early 1990s before a sharp and sustained decline in the late 1990s. Most people currently injecting drugs in Australia are part of a cohort that began injecting during the 1980s and 1990s. As a result, the population of people currently injecting drugs in Australia is older and has been injecting drugs for a longer duration than in 2000.

**Implications of all the available evidence**

There has been a decline in the number of people initiating injecting drug use since the 1990s in both Australia and the UK. Given evidence that the age of people who inject drugs is also increasing in other high-income countries (particularly in Europe), it appears there may have been a cultural shift away from injecting drug use across multiple countries. Moreover, investment in harm reduction programs in these countries (e.g., needle-syringe programs, opioid agonist treatment, hepatitis C treatment, overdose prevention) may be reducing mortality among this population. Where data allow, we encourage investigation of the increasing age of people who inject drugs in other countries to determine whether this phenomenon has occurred elsewhere. Our results suggest that healthcare provision for this population must consider the effects of ageing and long-term injecting drug use, including chronic disease, injecting-related injuries and diseases, sequelae of bloodborne viral infections, and premature cognitive decline.

## Introduction

The age of people who inject drugs is increasing in some high-income countries,^1^, ^2^, ^3^ which has implications for provision of healthcare to this population.^4^^,^^5^ A recent study using data from surveys with people who inject drugs proposed several explanations for the increasing age of this population in the UK.^3^ Firstly, the age at which people initiate injecting drug use may be increasing. Secondly, people may be injecting for longer periods of time, possibly because scale-up of interventions (e.g., needle-syringe programs, naloxone, HIV and hepatitis C virus treatment) has reduced morbidity and mortality among this population.^6^ Finally, it may be the result of a cohort effect, whereby the increasing age of this population is the result of a large group injecting drugs for the first time at a similar time. The latter of these was determined to be the most likely explanation for the ageing population in the UK, with a large cohort initiating injecting drug use in the 1990s and continuing to inject to this day.^3^

A similar trend appears to be evident in Australia. The age of people who report recent injecting drug use in national household surveys,^7^ age of clients accessing treatment for heroin or amphetamines,^8^ and age of drug-induced death^9^ are all increasing. However, people who inject drugs tend to be underrepresented in household surveys^10^ while treatment, hospitalisation and death data are likely skewed towards people who have been using drugs for a longer period of time and are thus older. Surveys that recruit people currently injecting drugs would provide a more representative insight into these population trends. Drug market shifts might offer an explanation in addition to those proposed by Lewer and colleagues in the UK^3^ for changes to Australian population demographics. Disruptions to the Australian heroin supply beginning in 2001 were believed to have reduced the number of people initiating injecting heroin use.^11^ A subsequent increase in availability and use of crystal methamphetamine,^12^ which is more typically smoked in Australia, may also have reduced injecting initiation. Examining changes to age, age of injecting drug use initiation (hereafter ‘initiation’), drug injected at initiation, and time since initiation in a setting with different drug markets and cultural factors may determine whether the cohort effect observed in the UK is evident more broadly.

The Illicit Drug Reporting System (IDRS) and Australian Needle and Syringe Program Survey (ANSPS) are national Australian surveillance mechanisms that conduct annual surveys with people currently injecting drugs, providing ideal data sources to examine trends in population characteristics.^13^^,^^14^ Using data from 2000 to 2019 surveys, we aimed to:

1. Describe trends in the current age, age of initiation, drug injected at initiation, and time since initiation.
2. Explore injecting drug use initiation ‘cohort’ effects.
3. Model the number of people initiating injecting drug use over time.

## Methods

### Study design and data source

This was a repeated cross-sectional study using data from two Australian national surveillance systems that conduct annual surveys with people who inject drugs: the IDRS and ANSPS. Eligibility criteria and participant recruitment strategies differ between the surveys but have remained consistent over time, meaning they both provide an opportunity to investigate changes in the demographic characteristics, including age, of people who inject drugs. We chose to use both surveys to improve the robustness of our findings.

The IDRS (2000 to present) monitors trends in illicit drug use, harms, and markets and includes annual interviews with a sentinel sample of people who regularly inject drugs (target annual sample size N = 900). Participants are recruited from each capital city of Australia via needle-syringe programs, treatment providers, and word-of-mouth. Eligibility criteria are: ≥17 years of age, injection of illicit or non-prescribed drugs (not including performance and image enhancing drugs) ≥ six times in the past six months, and residence in the city of interview for at least 10 of the past 12 months. Approximately 20% of people each year report participation in a previous year.

The ANSPS (1995 to present) recruits 2000–2500 attendees from more than 50 needle-syringe programs across Australia (both city and regional areas) during a two-week period each year. All needle-syringe attendees are eligible for participation. Participants complete a brief self-administered survey and provide a dried blood spot sample, which is tested for antibody to HIV and hepatitis C and hepatitis C RNA. ANSPS has been shown to be representative of the broader population of needle-syringe program attendees.^15^ Between 15 and 30% of participants report previous participation each year.

Further information about both the IDRS and ANSPS is provided in the Appendix (p3–6).

### Variables

The outcomes of interest were current age, birth year, age of initiation, drug injected at initiation (IDRS only, categorised as heroin, meth/amphetamine, pharmaceutical opioids [including opioid agonist treatment], and other), calendar year of initiation, and time since initiation. Participants in both the IDRS and ANSPS reported their current age and age of initiation; ANSPS participants also reported their birth year, while this was calculated for IDRS participants (i.e., survey year−current age). The remaining outcomes (i.e., calendar year of initiation and time since initiation) were calculated for all participants (e.g., time since initiation = current age−age of initiation).

Other variables used to describe the samples were gender identity, frequency of injecting drug use during the month prior to interview, drug injected most frequently in the past month (IDRS) or the last drug injected (ANSPS), both categorised as for drug injected first, current opioid agonist treatment (methadone, buprenorphine [sublingual or long-acting injection], or buprenorphine-naloxone), and jurisdiction (i.e., Australian state or territory) of interview. All data were self-reported.

### Analysis

We analysed data from 2000 to 2019 for both surveys. In 2020, some IDRS survey questions (including age of initiation) were removed to facilitate the addition of COVID-19 items. The impacts of the COVID-19 pandemic on service access affected participant recruitment in 2020 and 2021 for both surveys. Therefore, we chose to truncate the study period at 2019, rather than include the most recent data available.

To facilitate comparison between the two studies, we excluded ANSPS participants who reported that the most recent drug they injected was a performance and image enhancing drug (which does not count towards the eligibility criteria for the IDRS), and participants from either survey who did not report injecting drug use in the month prior to interview. We retained participants who reported participation in prior surveys as their increasing age should contribute to the overall age of the group. We excluded participants with missing data for any of the outcome variables.

To examine changes in survey demographics over time, we estimated the following quantile regression models and described results as the median and interquartile range [IQR], with the 95% confidence intervals and model coefficients reported in the Appendix (p7–9):

1. Current age of participants as the dependent variable and survey year (included as linear and quadratic terms) as the independent variable.
2. The age of initiation as the dependent variable and calendar year of initiation (included as linear and quadratic terms) as the independent variable. We restricted this analysis to participants who reported injecting drugs for the first time within three years of the survey. Because people who began injecting drugs at a younger age are more likely to remain in the population (and thus, remain eligible for the studies) for longer, using the whole sample for analysis may underestimate the true age of initiation.
3. Time since initiation as the dependent variable and survey year (linear and quadratic terms) as the independent variable.

We also described the distributions of birth year, time since initiation, and calendar year of initiation, using histograms stratified by survey year.

To model the number of people in Australia initiating injecting drug use each year, we first calculated the pairwise ratio of each combination of calendar year of initiation within each survey year (limiting to years of initiation from 1980 onwards). This allowed us to estimate the relative number of people beginning to inject drugs each year. Due to cessation of injecting or death, people may have exited the population between the two observed years in each pair. To adjust for this, we assumed the mean duration of injecting drug use was 15 years^3^ and used an exponential decay function to estimate the proportion of people who commenced injecting in the earlier year and remained in the population in the later year. We used bootstrapping with 1000 resamples to calculate the variance of each ratio and the combined data across survey years using inverse variance weighting. Monte Carlo simulations were used to estimate the ratio of new initiators in each year, relative to 1980. We then used an existing population size estimate of people in Australia who inject drugs in 2014^16^ to convert the relative numbers of people initiating drug injection each year to absolute numbers. We chose this population estimate for the main analysis as it disaggregated estimates by gender and jurisdiction, allowing us to conduct subgroup analyses. Further information about the modelling process is provided in the Appendix (p16–21).

To describe patterns in the drug injected at initiation, we applied the proportion of IDRS participants reporting each drug to the modelled number of people initiating injecting drugs each year. This allowed us to discern temporal trends in the drug injected first, relative to the absolute size of people initiating injecting drug use each year.

All analyses were conducted using R (version 4.3.1).

### Subgroup analyses

We disaggregated analyses by gender (due to differences in age of drug-induced death by gender^9^), drug injected first, and jurisdiction (due to differences in drug markets).

### Sensitivity analysis

For the model of the number of people beginning to inject drugs each year, we conducted a sensitivity analysis, varying the assumption of the mean duration of injecting drug use (from 10 to 20 years).

### Ethics

Ethics approval for the IDRS was obtained from the South Eastern Sydney Local Health District Human Research Ethics Committee and jurisdictional human research ethics communities where appropriate. Ethical approval for the ANSPS was provided by the Sydney Local Health District Ethics Review Committee (RPAH Zone) under the National Mutual Acceptance scheme (2019/ETHO7546), and the University of Tasmania Human Research Ethics Committee (H0017666), with site-specific and external entity approvals obtained for all participating sites.

### Role of the funding source

The funders of the study had no role in study design, data collection, data analysis, data interpretation, or writing of the report.

## Results

**Fig. 1:**
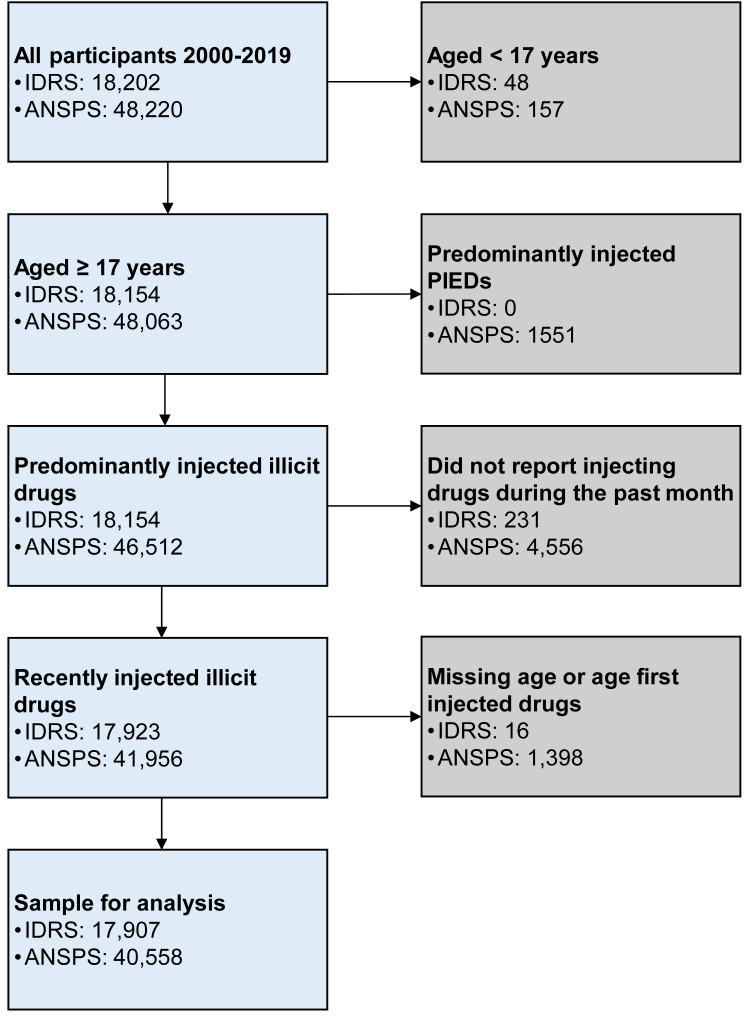
Participant flowchart. Notes: IDRS, Illicit Drug Reporting System; ANSPS, Australian Needle Syringe Program Survey; PIED, performance or image enhancing drug.

Between 2000 and 2019, 18,202 and 48,220 surveys were conducted in IDRS and ANSPS surveys, respectively. In total, data from 58,465 surveys were retained for analyses, comprising 17,907 from IDRS and 40,558 from ANSPS (Fig. 1). In both surveys, approximately two thirds of participants were men, half reported injecting drugs daily or more frequently in the past month, and just over a third reported current opioid agonist treatment (Table 1). More IDRS participants reported heroin (40.9%) than meth/amphetamine (30.5%) as the drug injected most frequently in the past month, while among ANSPS participants, similar numbers reported injecting these drugs most recently (35.0% and 34.2%).

**Table 1:** Characteristics of the IDRS and ANSPS samples, 2000–2019.

	IDRS	ANSPS	Total
**Total**	17,907 (100.0)	40,558 (100.0)	58,465 (100.0)
**Gender**			
Woman/female	6032 (33.7)	13,703 (33.8)	19,735 (33.7)
Man/male	11,822 (66.0)	26,599 (65.6)	38,421 (65.7)
Other^a^	39 (0.2)	0 (0.0)	39 (<0.1)
Missing	14 (0.1)	256 (0.6)	270 (0.5)
**State/territory**			
New South Wales	3019 (16.9)	11,410 (28.1)	14,429 (24.6)
Victoria	2994 (16.7)	6145 (15.2)	9139 (15.6)
Queensland	2043 (11.4)	10,161 (25.1)	12,204 (20.8)
Western Australia	1842 (10.3)	4232 (10.4)	6074 (10.4)
South Australia	2004 (11.2)	4544 (11.2)	6548 (11.2)
Tasmania	1985 (11.1)	1520 (3.7)	3505 (6.0)
Northern Territory	2034 (11.4)	1114 (2.7)	3148 (5.4)
Australian Capital Territory	1986 (11.1)	1432 (3.5)	3418 (5.8)
**Frequency of injecting drug use during past month**			
<Daily	9643 (53.9)	18,186 (44.8)	27,829 (47.5)
≥Daily	8264 (46.1)	22,372 (55.2)	30,636 (52.4)
**Main drug injected during past month**^b^			
Heroin	7329 (40.9)	14,188 (35.0)	21,517 (36.7)
Meth/amphetamine^c^	5465 (30.5)	13,888 (34.2)	19,353 (33.1)
Pharmaceutical opioid	4259 (23.8)	8630 (21.3)	12,889 (22.0)
Other	834 (4.7)	3852 (9.5)	4686 (8.0)
Missing	20 (0.1)	0 (0.0)	20 (<0.1)
**Current opioid agonist treatment**			
No	11,799 (65.9)	24,834 (61.2)	36,633 (62.7)
Yes	6088 (34.0)	15,724 (38.8)	21,812 (37.3)
Missing	20 (0.1)	0 (0.0)	20 (<0.1)
**Year of survey**			
2000–2004	4608 (25.7)	10,943 (27.0)	15,551 (26.6)
2005–2009	4505 (25.2)	9227 (22.8)	13,732 (23.5)
2010–2014	4405 (24.6)	10,020 (24.7)	14,425 (24.7)
2015–2019	4389 (24.5)	10,368 (25.6)	14,757 (25.2)

a Non-binary gender was not captured in ANSPS survey.

b IDRS participants reported main drug injected, ANSPS participants reported last drug injected.

c Includes amphetamine, methamphetamine powder (‘speed’), methamphetamine paste (‘base’), and crystal methamphetamine (‘ice’).

### Age of people who inject drugs

**Fig. 2:**
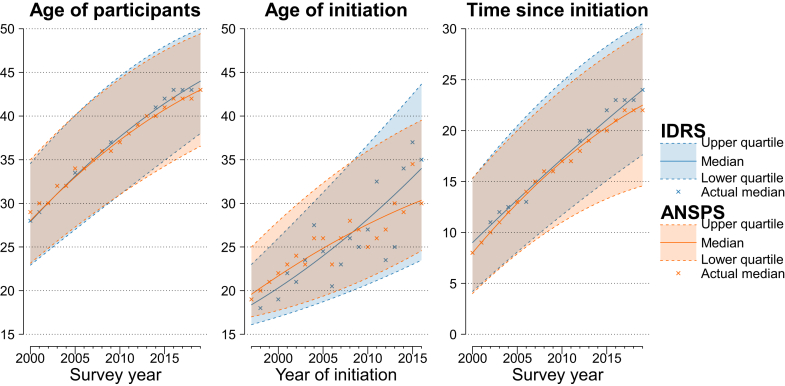
Age of IDRS and ANSPS participants (by survey year), age of injecting drug use initiation (by year of initiation), and time (in years) since injecting drug use initiation (by survey year). Notes: The median and interquartile range for each outcome were estimated using quantile regression with year as the independent variable (included as linear and quadratic terms). This figure is presented disaggregated by gender/sex, jurisdiction, and drug injected first in the Appendix (p10–14). Age of initiation analyses were limited to participants who reported initiation within three years of the survey; results without this restriction are provided in the Appendix (p15).

In both surveys, the median age of participants increased over the study period (Fig. 2, left panel). The median age for IDRS participants was 28 years (IQR: 23–35) in 2000 and 44 years (38–50) in 2019; for ANSPS participants this was 28 years (23–35) and 43 years (37–49), respectively. The increase in median age was consistent by gender and drug injected first, although the median age of men was consistently higher than that of women (Appendix p10) and higher among those who injected heroin first than those who injected meth/amphetamine first (Appendix p11). Results by jurisdiction are provided in the Appendix (p12).

### Age of injecting drug use initiation

The age of initiation increased with calendar year of initiation (Fig. 2, middle panel). Among those who initiated injecting drug use within three years of the survey, the median age of initiation for those who initiated in 1997 was 18 (16–23) in IDRS and 20 (17–25) in ANSPS, while for those who initiated in 2016, the median age was 34 (23–44) and 30 (25–40). The increase was consistent among men and women (Appendix p10) and among those who injected methamphetamine at initiation (Appendix p11). Results by jurisdiction are provided in the Appendix (p13). When the analysis was not restricted to those who began injecting within three years of the survey year, the increase in age of initiation was still evident from 1997 to 2016, but it was relatively stable prior to 1997 (Appendix p15).

### Year of injecting drug use initiation

**Fig. 3:**
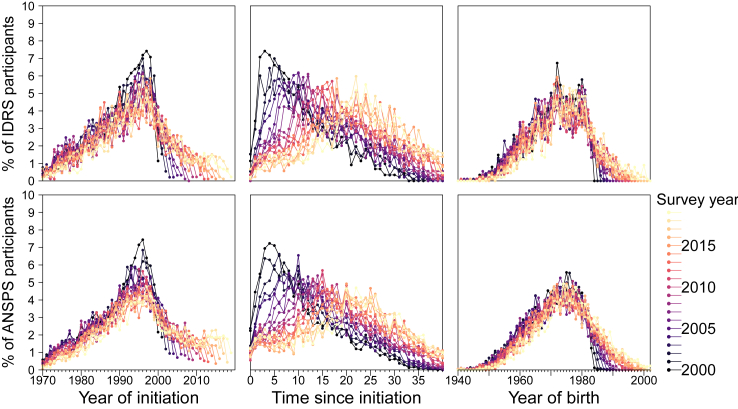
Year of birth, time since injecting drug use initiation, and year of initiation by survey year among IDRS (top) and ANSPS (bottom) participants.

Injecting initiation increased steadily from the 1970s to the mid-1990s, before declining. This observation was consistent irrespective of the survey year (Fig. 3, left panel).

### Time since injecting drug use initiation

Accordingly, time since initiation use increased across the study period (Fig. 2, right panel). In 2000, the median time since initiation was 8 (4–15) years in IDRS and 9 (4–15) years in ANSPS, which increased to 22.5 (18–31) and 24 (15–30) years in 2019, respectively. The increase was observed among both men and women, although men consistently reported longer time since initiation than women (Appendix p10). Time since initiation was similar by drug injected first in the first five years of the study period and increased more steeply among those who reported heroin as the drug injected first compared to those who reported meth/amphetamine (Appendix p5). Jurisdiction-level results are provided in the Appendix (p14).

The distribution of time since initiation was positively skewed in earlier years of the surveys with a peak at approximately five years (despite the median being 8–9 years; Fig. 3, middle panel). Over the survey period, the distribution flattened.

### Birth year of participants

Aligning with the increasing age of participants, there was a steady increase that peaked in the mid-1970s before steeply decreasing (Fig. 3, right panel). This pattern was evident in both surveys, although it was more consistent across survey years in the ANSPS.

### The estimated number of people initiating injecting drug use in Australia between 1980 and 2019

**Fig. 4:**
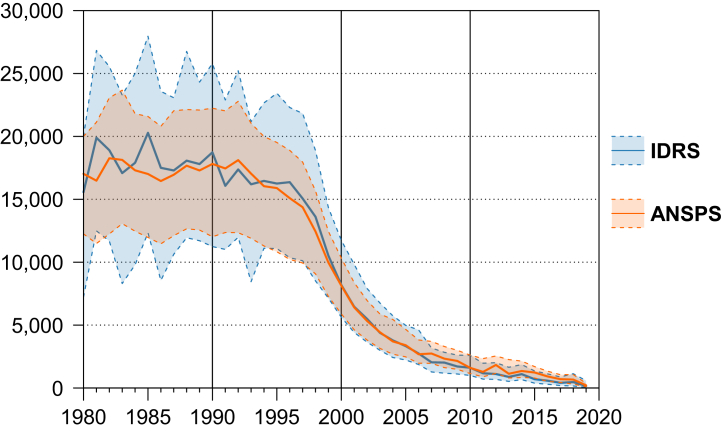
Modeled number of people initiation injecting drug use in Australia, with 95% prediction intervals. Notes: The number of people initiating injecting drug use each year was calculated by applying the relative size of new cohorts of people who inject drugs to an existing Australian population size estimate. Results by gender and jurisdiction are provided in the Appendix (p26 and 27).

Modelling suggests the number of people initiating injecting drug use each year was relatively stable from 1980 to the mid-1990s before a steep decrease was observed until the mid-2000s, after which initiations continued to decrease less rapidly (Fig. 4). Using IDRS survey data, we estimate the number of people initiating injecting drug use in 1980 was 15,570 (95% prediction interval 7240–20,000) and 16,360 (10,360–22,300) in 1996, before declining to 1110 (680–2010) in 2012 whereafter it remained low but fluctuated (Appendix p22 and 23). Using ANSPS survey data, we estimated the corresponding numbers to be 17,020 (12,250–20,000) in 1980, 15,090 (10,220–18,890) in 1996, and 1830 (1160–2540) in 2012 (Appendix p24 and 25). Prior to the decline in the mid-1990s, it appears that the number of men beginning to inject drugs was decreasing modestly, while the number of women was slightly increasing (Appendix p26). This aligns with the gap observed in time since initiation between men and women. Different patterns were observed across jurisdictions (Appendix p27).

**Fig. 5:**
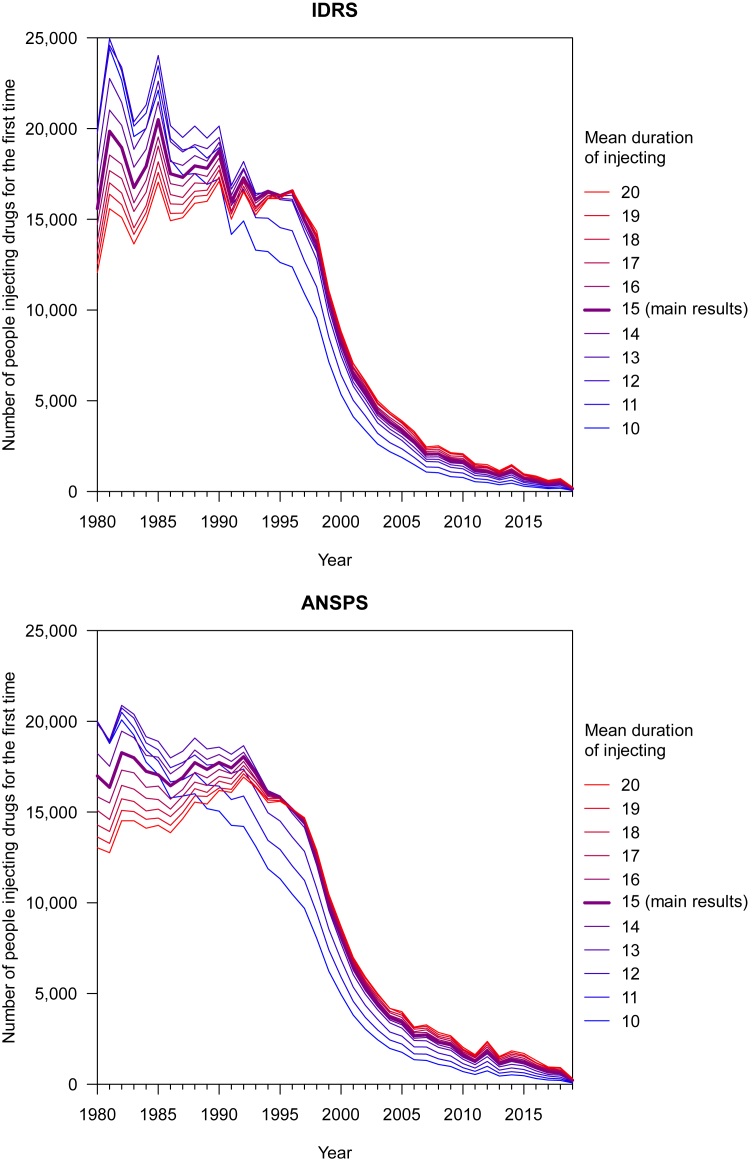
Sensitivity analysis of the effect of altering the assumed mean duration of injecting drug use on the modelled number of people injecting drugs for the first time, using IDRS (top) and ANSPS (bottom) data.

The shape of the curve prior to the decrease in the mid-1990s was sensitive to the assumption of mean duration of injecting used in the model (Fig. 5). When the assumed mean was larger (i.e., >15 years), the estimated number of people initiating injecting drug use increased from 1980 to the mid-1990s, however when the mean was smaller (i.e., <15 years) the estimated number was decreasing prior to the 1990s. The decrease in the mid-1990s was consistent irrespective of the assumed mean, although it is possible that this reduction started earlier.

**Fig. 6:**
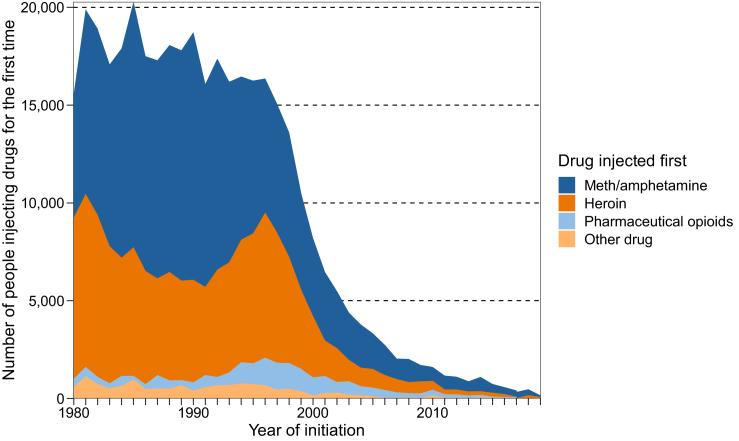
The number of people injecting drugs for the first time disaggregated by drug first injected, using the main model results for IDRS. Notes: The proportion of IDRS participants reporting initiation using each drug was applied to the modelled number of people initiating injecting drugs each year. Meth/amphetamine includes responses ‘amphetamines’, ‘liquid amphetamine’, ‘crystal methamphetamine (ice)’, ‘methamphetamine powder (speed)’, and ‘methamphetamine paste (base)’. Pharmaceutical opioids include opioid agonist treatment. Drug injected first data were not collected in ANSPS.

Examination of the drug first injected by year of initiation shows that initiating injecting with meth/amphetamine was most common across the survey period (Fig. 6). Initiating injecting with heroin declined between 1980 and 1990 before peaking in the mid-1990s and declining again thereafter, consistent with the overall decline in initiation. While relatively rare, injecting pharmaceutical opioids first was most common in the mid-to-late-1990s.

## Discussion

Using 20 years of data from two annual national Australian surveys with people who inject drugs (IDRS and ANSPS), we examined trends in age and injecting initiation. Trends observed between the two surveys were consistent. We demonstrated a sharp and sustained decline in the number of people initiating injecting drugs in the mid-1990s. Our findings suggest that most people currently injecting drugs in Australia were part of a cohort that began injecting in the 1980s or 1990s and as a result, the population in 2019 was older and had been injecting drugs for a longer period than the population who were injecting drugs in 2000 (as many were the same people). There was also an increase in age of initiation over time. Amphetamines were consistently reported by IDRS participants as the most common drug injected at initiation, although peaks in initiation with heroin were observed in the early 1980s and mid-1990s.

We cannot say for certain what is driving the observed decline in injecting drug use incidence, however the ‘drug subculture’ theory may provide a useful lens for interpretation. This theory posits that sociocultural settings, rather than characteristics of the drug or individual using the drug, are the predominant drivers of drug use prevalence^17^ and describes ‘drug eras’, which are characterised by incubation, expansion, plateau and decline phases. Our data suggest that this era of injecting drug use began expanding in the early 1970s, before plateauing between the 1980s and 1990s (the exact timing of the plateau phase was sensitive to model assumptions) and declining in the mid-to-late-1990s. People who are teenagers or young adults during the plateau phase typically make up the core population of the era,^17^ which is consistent with our observation that injecting drug use increased from birth cohorts born in the 1950s to those born in the early 1970s.

Counter to our expectations, the decline phase of injecting drug use initiation began in 1997 during a period marked by cheap and pure heroin,^18^^,^^19^ street-based heroin markets in Sydney^20^ and Melbourne,^21^ increasing numbers of people regularly using heroin,^22^ and elevated harms, including overdose.^22^ These harms and behaviours then declined after a disruption to heroin supply in late 2000.^22^ The lag between the decline in initiation and decline in related outcomes may be a result of people who injected amphetamines first and transitioned to heroin,^23^^,^^24^ or the time taken to progress from initiation to regular injecting (which ranged 1–4 years among a Melbourne sample during this time^25^). The drug subculture theory suggests that forms of drug use eventually go ‘out of favour’, with young people beginning to use drugs during this time resisting the norms from the generation before them, causing the decline phase.^17^ In the late 1990s there was an increase in visible (i.e., on the street, in the media) heroin-related harms and policing,^22^ which may have deterred people from initiating injecting use of heroin. However, initiation with amphetamines decreased simultaneously, pointing to a broader cultural shift away from injecting. Indeed, while the decrease in heroin availability and purity in late 2000 led to fewer people initiating injecting heroin use,^11^^,^^26^ markets eventually rebounded and heroin is perceived to be pure and easy to obtain today,^27^ but very few people are initiating injecting drug use. Although injecting drug use persists in Australia, it is largely among the cohort who began injecting in the 1980s or 1990s and continue to inject, perhaps due to substance dependence or entrenchment in the lifestyle or social aspect of injecting drugs.

Although much attention has been given to Australian heroin markets and harms in the literature,^28^ a key finding of this study is that amphetamines were the predominant driver of injecting drug use initiation in Australia over the past four decades. Drug markets have shifted in this time, with low purity amphetamine and methamphetamine powder available in the 1980s and 1990s before the proliferation of crystal methamphetamine in the 2000s.^29^ The higher purity of the crystal form means many people now smoke rather than inject methamphetamine,^7^^,^^30^ which may have a role in the sustained low incidence of injecting initiation. Despite the low numbers of people initiating injecting drug use with methamphetamine over the past two decades, methamphetamine-related harms have increased during this time.^29^ Recent analyses of people who were dependent on methamphetamine, which found no significant differences in non-injection related outcomes (e.g., frequency of use, violent behaviour, crime) between those who only smoked and those who only injected methamphetamine, leading the authors to caution against the assumption that smoking methamphetamine would lead to reduced harms.^31^ Collectively, these observations suggest that there is an increasing population of people using methamphetamine regularly or in a dependent manner (but not necessarily via injection) in Australia, and highlights the importance of investing in developing more effective treatment options and maximising methamphetamine treatment access and coverage.

Also of note were the gender differences we observed, with women who participated in the surveys tending to be younger and report initiation at a younger age. Moreover, it appears that the peak in incidence of initiation of injecting drug use occurred later than that for men. The younger age of initiation among women has been reported in other studies^32^^,^^33^ and an Australian study found that the time between first illicit drug use by any route of administration and injecting initiation was shorter for women than men.^34^ Other gender differences in initiation have previously been noted, for example, women are more likely to be injected by their partner.^33^^,^^35^, ^36^, ^37^ While we cannot be certain what caused the differences we observed, our findings provide further evidence for the role of gender in injecting initiation.

Overall, our observations align with previous UK analyses,^3^ although there was a more prominent peak of injecting drug use initiation in the mid-1990s in the UK and the subsequent decline was less steep than we estimated in our study. This consistency is noteworthy given differing drug markets and drug subcultures. Canada and some European countries have also reported that the age of people who inject drugs is increasing,^2^^,^^38^ potentially suggesting that this is a widespread phenomenon, at least in high income countries. Indeed, a systematic review found that increased median age and time since initiation is associated with higher country GDP.^39^ In contrast, while there was evidence that the population injecting drugs in the US was ageing in the early 2000s,^40^ more recent data suggest that injecting drug use prevalence has increased and people aged 18–39 years are overrepresented among this population.^41^ Increased pharmaceutical opioid use during the late 1990s and early 2000s in the US coincided with the decline in injecting drug use in the UK and Australia, which may have led to these disparate population trends. If current trends were to continue, our findings suggest there would eventually be very few people injecting drugs in Australia. Indeed, national household survey data suggests that the population of people who have injected drugs in the past year has declined over the past two decades (although these data are likely to be biased by underreporting, which may have changed over time).^7^ However, the reversal in trend in the US is a reminder that there may be future injecting drug use eras in Australia and other high-income countries. Ongoing surveillance, through surveys that recruit people actively injecting drugs like the IDRS and ANSPS, provide mechanisms to monitor this, for example, by examining changes in the proportion of participants who recently initiated injecting drug use.

Our observations have policy implications particularly with respect to healthcare provision. Illicit drug use is associated with an accelerated physiological ageing process, meaning chronic cardiac, pulmonary, kidney and liver conditions may occur earlier in life^42^ and might be more prevalent compared to the general population.^43^ People who have injected drugs for a longer time are also more likely to have been exposed to blood-borne viral infections such as hepatitis C^44^ and are at increased risk of injecting related injuries due to poor venous conditions.^45^ Many will also be living with the effects of chronic poverty, poor nutrition, and limited preventive care on their health. Isolation and stigma associated with drug use have consequences for ‘successful ageing’,^4^ which includes sustained engagement in social activities,^46^ and create barriers to drug treatment access and retention.^47^ Potential drug interactions between OAT and medications for chronic circulatory and respiratory conditions mean care should be taken in the medicine prescribed to this group.^48^^,^^49^ There is an urgent need to increase the capacity of primary and geriatric care sectors (including aged care facilities) to provide appropriate care to older people who inject drugs as they are likely to have increasing contact with this population. However, there is currently an absence of primary care models and chronic disease care models that are specific to this population.^4^

### Limitations

We note five limitations with respect to generalisability of the trends observed in this study. First, all ANSPS, and most IDRS participants, were recruited from services, which may mean people who have more recently commenced injecting drugs are underrepresented. Second, in both surveys, people can participate regardless of participation in previous years, which may have contributed to the increasing age and time since initiation we observed. However, restricting the samples to people who had never participated before might bias the sample towards people who are younger and/or more recently commenced injecting. Further, an analysis of representativeness of the ANSPS survey found that people who were older and injected heroin were not preferentially recruited relative to those who were younger and injected methamphetamine.^15^ Third, the IDRS recruits people who inject drugs regularly (≥monthly), so findings may not be representative of all people currently injecting drugs. Nevertheless, results using IDRS data were similar to those using ANSPS data which has no eligibility criteria based on injecting frequency. Fourth, people can participate in both surveys and some recruitment sites are shared between IDRS and ANSPS, which may have contributed to the consistency observed between studies. However, ANSPS has wider geographic coverage with many more recruitment sites, including outside of capital cities. Fifth, our study period ended prior to the COVID-19 pandemic which caused disruptions to the Australian heroin and methamphetamine supply^50^; this may have affected initiation to injecting drug use.

We also note some limitations of the analyses. The decision to retain repeat participants means there was non-independence of some observations in the quantile regression models, although previous research using the IDRS data found that non-adjustment for this issue is unlikely to affect population inferences, including for age.^51^ We relied on self-report data, which may be subject to recall bias particularly for those who initiated injecting drug use many years prior to the survey. Modelling results were dependent on an existing population size estimate calculated using a multiplier method,^16^ which has limitations.^52^ Use of another Australian population size estimate calculated using the same method with different benchmark data^53^ would have rescaled our results.

### Conclusion

Our findings demonstrate that people who currently inject drugs in Australia are older and have been injecting drugs for a longer period of time than people who injected drugs in 2000. Modelling suggests that this change in population characteristics is the result of a stable or increasing number of people beginning to inject drugs through the 1980s and early 1990s, which was followed by a sharp and sustained decline in initiation of injecting drug use. Our findings have implications for health service delivery to people who inject drugs, with an increase in age likely to be accompanied by a rise in chronic health conditions and an increase in injecting duration potentially resulting in increased exposure to blood-borne viruses and a higher incidence of injecting-related injury and disease.

## Contributors

OP and FZ prepared the dataset. OP analysed the data and produced the figures based on methodology developed by DL. OP drafted the manuscript. All authors made substantial contributions to critical review, editing, and revision of the manuscript. OP, LM, AP, FZ, BM and RS had access to the data used in the study. OP and FZ verified the data. All authors had final responsibility for the decision to submit the manuscript.

## Data sharing statement

Data were collected during interviews with consenting participants. The data are not publicly available due to ethical constraints.

## Declaration of interests

In the past 3 years, LD has received investigator-initiated untied educational grants for studies of opioid medications in Australia from Indivior. In the past 3 years, SL has received advisory board fees from Gilead Sciences. These companies and organisations had no knowledge of or role in the design, conduct, interpretation, or publication of these findings. All other authors declare no competing interests.

## Appendix A Supplementary data

Price Changes in age etc Supplementary revised
