# Supplementary material for: Changing age profile and incidence of injecting drug use initiation among people in Australia who inject drugs: evidence from two national repeated cross-sectional studies
Source: Lancet Reg Health West Pac. 2025 Apr 17;57:101548. doi: 10.1016/j.lanwpc.2025.101548 (PMC12036076; doi:10.1016/j.lanwpc.2025.101548)

Population ageing and apparent decline in initiation of injecting drug use in Australia

Supplementary material

Olivia Price, Lisa Maher, Amy Peacock, Farzaneh Zolala, Louisa Degenhardt, Paul Dietze, Sarah Larney, Seraina Agramunt, Samantha Colledge-Frisby, Bradley Mathers, Dan Lewer, Rachel Sutherland

Appendix A. Information about the IDRS and ANSPS. 3

Appendix B. The estimated median and interquartile range of age, age at initiation, and time since injecting drug use initiation among IDRS and ANSPS participants. 7

Appendix C. Age, age at initiation, and time since injecting drug use initiation among IDRS and ANSPS participants, disaggregated by gender. 10

Appendix D. Age, age at initiation, and time since injecting drug use initiation among IDRS participants, disaggregated by drug injected first. 11

Appendix E. Age, age at initiation, and time since injecting drug use initiation among IDRS and ANSPS participants, disaggregated by jurisdiction. 12

Appendix F. Age of initiation among IDRS and ANSPS participants, without restricting the sample to those who initiated injecting drug use within 3 years of the survey. 15

Appendix G. Model information. 16

Appendix H. The modelled number of people injecting drugs for the first time in Australia. 22

Appendix I. Modelled number of men and women injecting drugs for the first time in Australia. 26

Appendix J. Modelled number of people initiating injecting drug use in each Australian jurisdiction. 27

Appendix A. Information about the IDRS and ANSPS.

Illicit Drug Reporting System

Background

The Illicit Drug Reporting System (IDRS) is a monitoring system identifying trends in illicit drug markets in Australia. It was established to provide a coordinated approach to the monitoring of the use of illicit drugs, in particular, heroin, amphetamine, cocaine and cannabis. The IDRS is an ongoing project that has been conducted on an annual basis in Sydney, New South Wales since 1996, and in all capital cities of Australia since 2000.

As the purpose of the IDRS was to detect emerging trends in illicit drug use of potential national importance, data collection for the IDRS was restricted to capital cities. Capital cities contain the major drug markets (e.g., the Melbourne suburb of North Richmond) wherein the majority of drug use occurs. As such, it is in these cities that new trends, that may diffuse to other areas, are likely to emerge. The IDRS monitors the price, perceived purity and perceived availability of heroin, methamphetamine, cocaine, cannabis and other drugs. It also examines trends in the use of these drugs, and associated behaviours and harms. It does this via analyses of data from interviews with people who regularly inject illicit drugs, as well as other routinely collected indicator data sources. The IDRS is designed to be sensitive to emerging trends, providing data in a timely manner, rather than describing issues in extensive detail.

The aims of the IDRS interview component are to:

1. Describe the characteristics of a sample of people who regularly inject illicit drugs, interviewed in each capital city of Australia;
2. Examine the patterns of drug use among this sample;
3. Document the current price, perceived purity and perceived availability of illicit drugs in the capital cities of Australia;
4. Examine participants' reports of drug-related behaviours (e.g., harm reduction behaviours) and harm, including physical, psychological, occupational, social and legal harms; and
5. Identify emerging trends in the illicit drug market that may require further investigation.

Methodology

Since 2000, the sentinel population chosen for interviews has consisted of people who report regularly injecting illicit or non-prescribed drugs. The IDRS is primarily concerned with four main drug classes: heroin, methamphetamine, cocaine and cannabis. It also monitors the use of pharmaceutical opioids and other drugs, as well as issues related to drug use (e.g., injecting-related injuries and non-fatal overdose).

The methodology for the IDRS is kept consistent each year for the purpose of studying trends. Where possible, recruitment occurs through the same sites (i.e., treatment agencies and needle syringe programs (NSP)) each year as it is imperative that there is consistency in recruitment methods from year to year for comparison.

The recruitment method is consistent over the period of monitoring. Participants are recruited through a purposive sampling strategy, mostly through treatment agencies, needle and syringe programs (NSP) and 'snowball' procedures. 'Snowballing' is a means of sampling hidden

populations which relies on peer referral, and is widely used to access people who use illicit drugs both in Australian and international studies. On completion of the interview, participants are asked if they would be happy to discuss the study with friends who might be willing and able to participate.

Given the emergence of COVID-19 and the resulting restrictions on travel and people's movement in Australia (which first came into effect in March 2020), face-to-face interviews were not always possible due to the risk of infection transmission for both interviewers and participants. For this reason, all methods from 2020 and onwards were similar to previous years, with the exception of changes in some means of recruitment, data collection, and reimbursement.

The participants

Many of the sociodemographic characteristics of the sample have largely similar over the study period. Typically, two thirds of participants identify as men, the average years of school completed is 10, approximately one tenth report completion of a university degree, and between one third and one half are in drug treatment at the time of interview. Age, the proportion of the sample identifying as Aboriginal and/or Torres Strait Islander, the proportion reporting lifetime incarceration and the proportion reporting current unemployment have increased over the study period.

Further information can be found here:

Sutherland, R., et al. (2024). "Illicit Drug Reporting System (IDRS) Interviews 2024: Background and Methods." Sydney: National Drug and Alcohol Research Centre, UNSW Sydney.

Australian Needle and Syringe Program Survey (ANSPS)

Background

The Australian Needle and Syringe Program Survey (ANSPS) functions as a strategic early-warning system designed to monitor blood borne viral infections and injecting and sexual behaviour among people who inject drugs. The ANSPS has been conducted annually in all Australian states and territories since 1995.

As a biobehavioural surveillance system, the ANSPS provides serial point prevalence estimates of HIV and hepatitis C virus antibody and RNA prevalence and sexual and injecting risk behaviour among people who inject drugs (PWID) in Australia.

NSP services were initially selected to participate in the ANSPS based on the number of client occasions of service per week, willingness to participate, and representation from all jurisdictions. Sample sizes have ranged from 1,072 (in 1995) to 2,742 (in 2018). In 2019, a total of 51 NSP services had participated in ten or more survey years; including 25 services that participated in 20 or more survey years and ten services that participated each year since 1995.

Methods

Staff at NSP services record the age and gender of both ANSPS respondents and refusals, as well as whether each individual is a new or repeat client. These records are used to calculate annual ANSPS response rates, with only one attendance per person included in the response rate denominator. Annual response rates have ranged from 38-60%. In 2006, additional response rate data were collected to enable a comparison of the demographic and drug use characteristics of respondents and non-respondents with a view to assessing the representativeness of the ANSPS survey sample. Topp et al., reported that females were more likely to agree to participate in the survey than males.[1] Nonetheless, the authors concluded that inferences derived from ANSPS samples can be reasonably applied to the broader NSP client population and that the ANSPS sample is as representative of Australia's PWID population as is practical to obtain.

During the survey implementation period, all NSP attendees are invited to participate in the ANSPS by NSP staff. Verbal rather than written consent is obtained, and participation is anonymous and voluntary. Consenting NSP attendees complete a brief self-administered questionnaire and provide a capillary dried blood spot. The questionnaire collects data on demographic characteristics, injecting and sexual behaviours, and history of BBV testing, imprisonment and drug treatment. Although the survey instrument has undergone minor modification over time and has periodically included additional modules to examine specific issues of interest, core elements and key questions have remained constant over the study period. The survey is designed to be self-completed, although assistance for clients is available where necessary.

Participants

Similar to the IDRS, in most years two thirds of respondents were male, while the median age of the sample and the proportion of respondents identifying as Aboriginal and/or Torres Strait

Islander over the survey period, and the proportion reporting lifetime incarceration all increased over the survey period.

Further information can be found here:

Heard, S., et al. (2020). "Australian NSP survey: Prevalence of HIV, HCV and injecting and sexual behaviour among NSP attendees, 25-year National Data Report 1995-2019." Sydney: Kirby Institute, UNSW Sydney.

References

1. Topp, L., et al. (2008). "Representativeness of injecting drug users who participate in HIV surveillance: results from Australia's Needle and Syringe Program Survey." J Acquir Immune Defic Syndr 47(5): 632-638.

Appendix B. The estimated median and interquartile range of age, age at initiation, and time since injecting drug use initiation among IDRS and ANSPS participants.

For brevity, only results for the entire samples are reported here. For results disaggregated by gender, jurisdiction, and drug injected first, please contact the corresponding author.

B1. Estimated age of IDRS and ANSPS participants.

	IDRS			ANSPS		
	**Median (95% CI)**	**Q1 (95% CI)**	**Q3 (95% CI)**	**Median (95% CI)**	**Q1 (95% CI)**	**Q3 (95% CI)**
**2000**	27.9 (27.4-28.4)	22.9 (22.5-23.3)	34.6 (34-35.1)	28 (27.8-28.2)	23.1 (22.9-23.4)	35 (34.7-35.3)
**2001**	29 (28.6-29.4)	23.7 (23.4-24)	35.7 (35.3-36.2)	29.1 (28.9-29.3)	24 (23.8-24.2)	36.1 (35.9-36.3)
**2002**	30.1 (29.7-30.4)	24.6 (24.3-24.8)	36.9 (36.5-37.3)	30.1 (29.9-30.3)	24.8 (24.7-25)	37.1 (37-37.3)
**2003**	31.1 (30.8-31.4)	25.4 (25.2-25.6)	38 (37.7-38.3)	31.1 (31-31.2)	25.7 (25.5-25.8)	38.2 (38-38.3)
**2004**	32.1 (31.9-32.4)	26.2 (26-26.4)	39.1 (38.8-39.4)	32.1 (31.9-32.2)	26.5 (26.3-26.6)	39.1 (39-39.3)
**2005**	33.1 (32.9-33.4)	27 (26.8-27.2)	40.1 (39.8-40.4)	33 (32.8-33.2)	27.3 (27.1-27.4)	40.1 (39.9-40.3)
**2006**	34.1 (33.8-34.3)	27.8 (27.6-28)	41.1 (40.8-41.4)	33.9 (33.7-34.1)	28.1 (27.9-28.2)	41 (40.8-41.2)
**2007**	35 (34.7-35.3)	28.6 (28.4-28.8)	42 (41.7-42.3)	34.8 (34.6-35)	28.8 (28.7-29)	41.9 (41.7-42.1)
**2008**	35.9 (35.6-36.2)	29.4 (29.2-29.7)	42.9 (42.6-43.2)	35.6 (35.5-35.8)	29.6 (29.4-29.7)	42.7 (42.5-42.9)
**2009**	36.8 (36.5-37.1)	30.2 (30-30.5)	43.7 (43.4-44.1)	36.5 (36.3-36.6)	30.3 (30.1-30.4)	43.5 (43.3-43.7)
**2010**	37.6 (37.3-37.9)	31 (30.7-31.3)	44.6 (44.2-44.9)	37.2 (37.1-37.4)	31 (30.8-31.1)	44.3 (44.1-44.5)
**2011**	38.4 (38.2-38.7)	31.8 (31.5-32)	45.3 (45-45.7)	38 (37.8-38.2)	31.7 (31.5-31.8)	45 (44.8-45.2)
**2012**	39.2 (39-39.5)	32.6 (32.3-32.8)	46.1 (45.8-46.4)	38.7 (38.6-38.9)	32.3 (32.2-32.5)	45.7 (45.5-45.9)
**2013**	40 (39.7-40.3)	33.4 (33.1-33.6)	46.7 (46.5-47)	39.4 (39.3-39.6)	33 (32.9-33.1)	46.3 (46.1-46.5)
**2014**	40.7 (40.5-41)	34.1 (33.9-34.4)	47.4 (47.1-47.7)	40.1 (39.9-40.3)	33.6 (33.5-33.8)	46.9 (46.8-47.1)
**2015**	41.4 (41.2-41.7)	34.9 (34.7-35.1)	48 (47.7-48.3)	40.7 (40.6-40.9)	34.3 (34.1-34.4)	47.5 (47.3-47.7)
**2016**	42.1 (41.8-42.4)	35.7 (35.5-35.9)	48.6 (48.3-48.9)	41.3 (41.2-41.5)	34.9 (34.7-35)	48 (47.9-48.2)
**2017**	42.8 (42.5-43.1)	36.5 (36.2-36.7)	49.1 (48.7-49.4)	41.9 (41.7-42.1)	35.4 (35.2-35.7)	48.5 (48.3-48.7)
**2018**	43.4 (43-43.8)	37.2 (36.9-37.6)	49.6 (49.1-50)	42.5 (42.2-42.7)	36 (35.7-36.3)	49 (48.8-49.2)
**2019**	44 (43.5-44.5)	38 (37.6-38.4)	50 (49.5-50.5)	43 (42.7-43.3)	36.5 (36.2-36.9)	49.4 (49.1-49.7)

Notes. CI = confidence interval; Q1 = first quartile (25th percentile); Q3 = third quartile (75th percentile).

B2. Coefficients for the quantile regression models estimating age as a function of survey year.

	IDRS			ANSPS		
	**Median**	**Q1**	**Q3**	**Median**	**Q1**	**Q3**
**Year**	56.7 (12.5)	7.2 (10.5)	84.5 (14.4)	60.6 (7.2)	36.2 (7.5)	75.2 (7.9)
**Year^2**	-0.01 (0.003)	-0.001 (0.003)	-0.02 (0.006)	-0.01 (0.002)	-0.009 (0.002)	-0.02 (0.002)

Notes. Reported as coefficient (standard error). Coefficients are reported as raw polynomials.

B3. Estimated age of initiation for IDRS and ANSPS participants.

	IDRS			ANSPS		
	Median (95% CI)	Q1 (95% CI)	Q3 (95% CI)	Median (95% CI)	Q1 (95% CI)	Q3 (95% CI)
**1997**	18.4 (17.6-19.1)	16.1 (15.5-16.7)	23 (21.2-24.8)	19.6 (18.8-20.3)	17 (16.5-17.5)	25 (24.1-25.9)
**1998**	19 (18.5-19.5)	16.4 (15.9-16.8)	24 (22.6-25.4)	20.3 (19.7-20.9)	17.2 (16.8-17.6)	26 (25.4-26.7)
**1999**	19.6 (19.1-20.2)	16.7 (16.3-17.1)	25 (23.9-26.1)	21 (20.5-21.5)	17.5 (17.2-17.8)	27 (26.5-27.5)
**2000**	20.3 (19.7-20.9)	17 (16.6-17.4)	26 (24.9-27.1)	21.7 (21.3-22.1)	17.7 (17.5-18)	28 (27.4-28.5)
**2001**	21 (20.2-21.8)	17.3 (16.9-17.8)	27 (25.9-28.2)	22.4 (21.9-22.8)	18 (17.7-18.3)	28.9 (28.3-29.5)
**2002**	21.7 (20.8-22.6)	17.7 (17.2-18.2)	28.1 (26.8-29.4)	23 (22.5-23.5)	18.3 (18-18.7)	29.8 (29.1-30.5)
**2003**	22.4 (21.4-23.5)	18 (17.4-18.6)	29.1 (27.7-30.6)	23.6 (23-24.2)	18.7 (18.3-19)	30.7 (29.9-31.4)
**2004**	23.2 (22.1-24.3)	18.4 (17.7-19)	30.2 (28.6-31.8)	24.3 (23.6-24.9)	19 (18.6-19.4)	31.5 (30.7-32.3)
**2005**	24 (22.8-25.2)	18.7 (18.1-19.4)	31.2 (29.6-32.9)	24.9 (24.2-25.5)	19.4 (18.9-19.8)	32.3 (31.5-33.2)
**2006**	24.8 (23.5-26)	19.1 (18.4-19.8)	32.3 (30.6-34)	25.4 (24.7-26.1)	19.7 (19.3-20.2)	33.1 (32.2-34)
**2007**	25.6 (24.3-26.9)	19.5 (18.8-20.2)	33.4 (31.7-35.1)	26 (25.3-26.7)	20.1 (19.7-20.6)	33.9 (33-34.8)
**2008**	26.4 (25.2-27.7)	19.9 (19.2-20.6)	34.5 (32.8-36.2)	26.6 (25.9-27.2)	20.6 (20.1-21)	34.6 (33.7-35.5)
**2009**	27.3 (26-28.6)	20.3 (19.6-21)	35.6 (33.9-37.3)	27.1 (26.4-27.8)	21 (20.5-21.5)	35.3 (34.4-36.2)
**2010**	28.2 (26.8-29.6)	20.7 (20-21.5)	36.7 (35.1-38.4)	27.6 (26.9-28.3)	21.4 (20.9-22)	36 (35.1-36.9)
**2011**	29.1 (27.6-30.6)	21.2 (20.3-22)	37.9 (36.2-39.5)	28.1 (27.4-28.8)	21.9 (21.3-22.5)	36.7 (35.7-37.6)
**2012**	30 (28.3-31.8)	21.6 (20.6-22.6)	39 (37.3-40.7)	28.6 (27.8-29.4)	22.4 (21.7-23.1)	37.3 (36.3-38.3)
**2013**	31 (29-33)	22.1 (20.9-23.2)	40.1 (38.2-42.1)	29.1 (28.1-30)	22.9 (22.1-23.8)	37.9 (36.7-39.1)
**2014**	32 (29.6-34.3)	22.5 (21.1-23.9)	41.3 (39.1-43.5)	29.5 (28.4-30.6)	23.5 (22.4-24.5)	38.5 (37.1-39.8)
**2015**	33 (30.2-35.7)	23 (21.3-24.7)	42.5 (39.8-45.1)	29.9 (28.6-31.2)	24 (22.7-25.3)	39 (37.4-40.6)
**2016**	34 (30.7-37.3)	23.5 (21.5-25.5)	43.7 (40.4-46.9)	30.4 (28.8-31.9)	24.6 (23.1-26.1)	39.5 (37.6-41.5)

Notes. CI = confidence interval; Q1 = first quartile (25th percentile); Q3 = third quartile (75th percentile).

B4. Coefficients (standard errors) for the quantile regression model estimating age of initiation as a function of year of initiation.

	IDRS			ANSPS		
	Median	Q1	Q3	Median	Q1	Q3
**Year**	-30.1 (2.2)	-15.7 (1.9)	-51.2 (2.5)	-73.7 (16.6)	-53.1 (13.4)	-2.40 (24.6)
**Year^2**	0.008 (0.0006)	0.004 (0.0005)	0.02 (0.0006)	0.02 (0.004)	0.01 (0.003)	0.001 (0.006)

Notes. Reported as coefficient (standard error). Coefficients are reported as raw polynomials.

B5. Estimated time since initiation among IDRS and ANSPS participants.

	IDRS			ANSPS		
	Median (95% CI)	Q1 (95% CI)	Q3 (95% CI)	Median (95% CI)	Q1 (95% CI)	Q3 (95% CI)
**2000**	9 (8.7-9.3)	4.2 (3.9-4.5)	15.3 (14.9-15.7)	8 (7.7-8.3)	4 (3.8-4.2)	15.3 (15-15.6)
**2001**	9.9 (9.6-10.1)	5 (4.7-5.3)	16.4 (16.1-16.7)	9 (8.8-9.2)	4.8 (4.7-5)	16.3 (16.1-16.6)
**2002**	10.7 (10.5-10.9)	5.8 (5.6-6)	17.5 (17.2-17.7)	10 (9.9-10.2)	5.7 (5.5-5.8)	17.3 (17.1-17.5)
**2003**	11.5 (11.3-11.7)	6.6 (6.4-6.8)	18.5 (18.3-18.7)	11 (10.8-11.2)	6.4 (6.3-6.6)	18.2 (18-18.4)
**2004**	12.4 (12.2-12.6)	7.3 (7.1-7.5)	19.5 (19.3-19.7)	11.9 (11.8-12.1)	7.2 (7.1-7.3)	19.1 (18.9-19.3)
**2005**	13.2 (13-13.4)	8.1 (7.9-8.3)	20.5 (20.2-20.7)	12.9 (12.7-13)	7.9 (7.8-8)	20 (19.8-20.2)
**2006**	14 (13.8-14.3)	8.8 (8.6-9)	21.4 (21.1-21.7)	13.7 (13.6-13.9)	8.6 (8.4-8.7)	20.9 (20.6-21.1)
**2007**	14.8 (14.6-15.1)	9.6 (9.3-9.8)	22.3 (22-22.6)	14.6 (14.4-14.8)	9.2 (9.1-9.4)	21.7 (21.5-21.9)
**2008**	15.6 (15.4-15.9)	10.3 (10-10.5)	23.2 (22.9-23.5)	15.4 (15.2-15.6)	9.9 (9.7-10)	22.5 (22.3-22.7)
**2009**	16.4 (16.2-16.7)	11 (10.8-11.2)	24 (23.7-24.3)	16.2 (16-16.4)	10.4 (10.3-10.6)	23.3 (23-23.5)
**2010**	17.2 (16.9-17.5)	11.7 (11.5-12)	24.8 (24.5-25.1)	17 (16.8-17.2)	11 (10.8-11.2)	24 (23.8-24.2)
**2011**	18 (17.7-18.3)	12.4 (12.2-12.6)	25.6 (25.3-25.9)	17.7 (17.5-17.9)	11.5 (11.4-11.7)	24.7 (24.5-24.9)
**2012**	18.8 (18.5-19)	13.1 (12.8-13.3)	26.3 (26-26.6)	18.4 (18.2-18.6)	12 (11.8-12.2)	25.4 (25.2-25.6)
**2013**	19.5 (19.3-19.8)	13.8 (13.5-14)	27 (26.7-27.3)	19.1 (18.9-19.2)	12.5 (12.3-12.7)	26.1 (25.9-26.3)
**2014**	20.3 (20.1-20.5)	14.4 (14.2-14.7)	27.7 (27.4-27.9)	19.7 (19.6-19.9)	12.9 (12.7-13.1)	26.7 (26.5-26.9)
**2015**	21.1 (20.8-21.3)	15.1 (14.8-15.4)	28.3 (28-28.6)	20.3 (20.2-20.5)	13.3 (13.1-13.5)	27.3 (27.1-27.5)
**2016**	21.8 (21.5-22.1)	15.7 (15.4-16.1)	28.9 (28.6-29.2)	20.9 (20.8-21.1)	13.7 (13.4-13.9)	27.9 (27.7-28.1)
**2017**	22.5 (22.2-22.9)	16.4 (16-16.8)	29.5 (29.1-29.8)	21.5 (21.3-21.7)	14 (13.7-14.3)	28.5 (28.2-28.7)
**2018**	23.3 (22.9-23.6)	17 (16.5-17.5)	30 (29.6-30.4)	22 (21.8-22.2)	14.3 (13.9-14.7)	29 (28.7-29.3)
**2019**	24 (23.6-24.4)	17.6 (17.1-18.2)	30.5 (30-31)	22.5 (22.2-22.8)	14.6 (14.1-15)	29.5 (29.2-29.8)

Notes. CI = confidence interval; Q1 = first quartile (25th percentile); Q3 = third quartile (75th percentile).

B6. Coefficients for the quantile regression model estimating time since initiation as a function of survey year.

	IDRS			ANSPS		
	**Median**	**Q1**	**Q3**	**Median**	**Q1**	**Q3**
**Year**	15.2 (10.7)	20.5 (11.4)	67.8 (12.5)	60.3 (7.3)	64.7 (8.3)	54.8 (8.8)
**Year^2**	-0.004 (0.003)	-0.005 (0.003)	-0.02 (0.003)	-0.01 (0.002)	-0.02 (0.002)	-0.01 (0.002)

Notes. Reported as coefficient (standard error). Coefficients are reported as raw polynomials.

Appendix C. Age, age at initiation, and time since injecting drug use initiation among IDRS and ANSPS participants, disaggregated by gender.

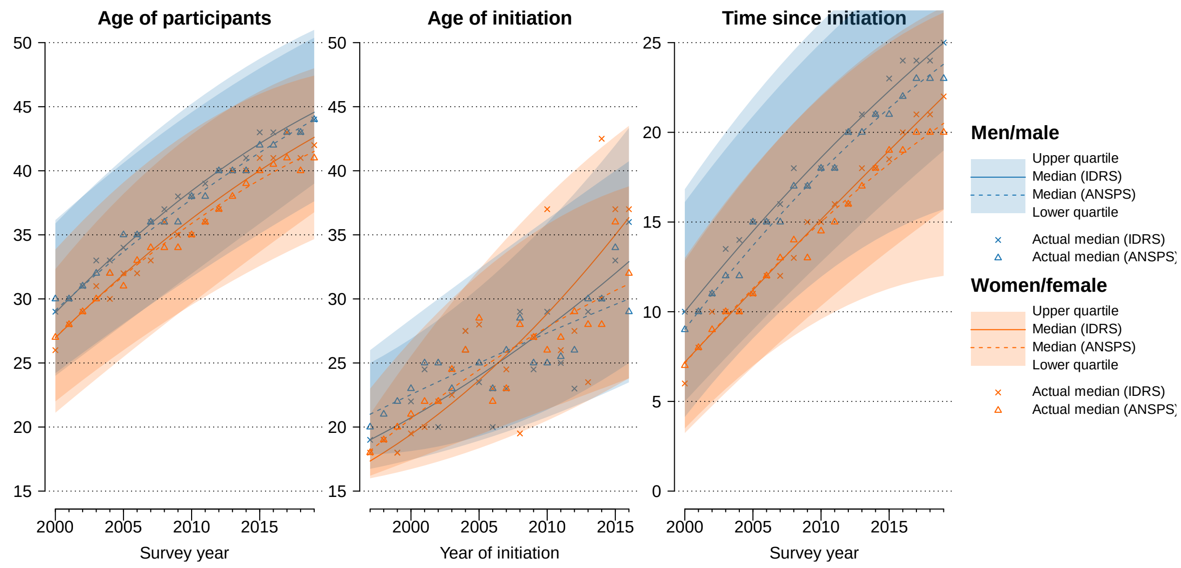

Appendix D. Age, age at initiation, and time since injecting drug use initiation among IDRS participants, disaggregated by drug injected first.

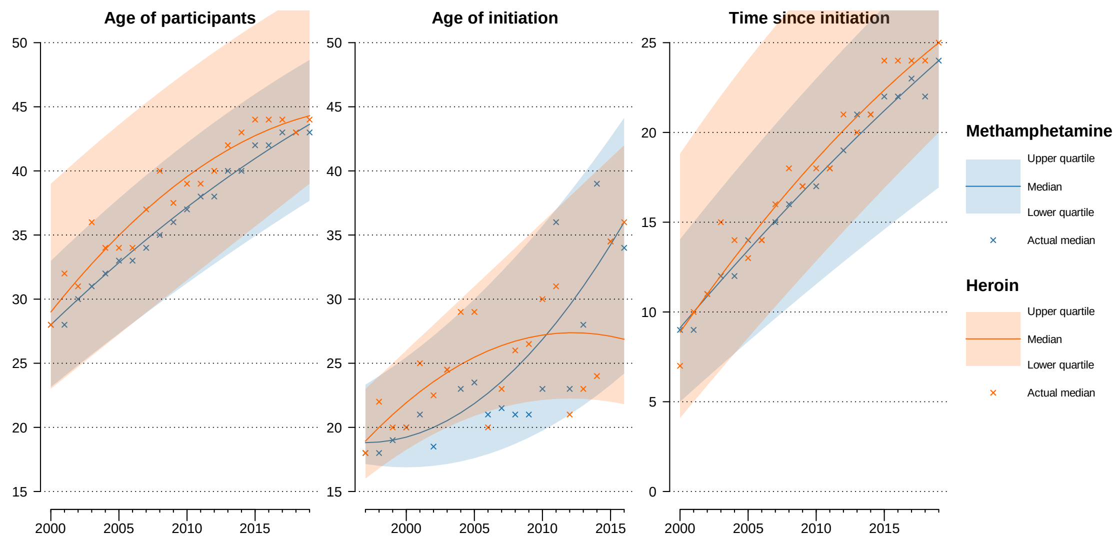

Notes. Drug injected first information not available for ANSPS participants.

Appendix E. Age, age at initiation, and time since injecting drug use initiation among IDRS and ANSPS participants, disaggregated by jurisdiction.

E1. Age of IDRS and ANSPS participants.

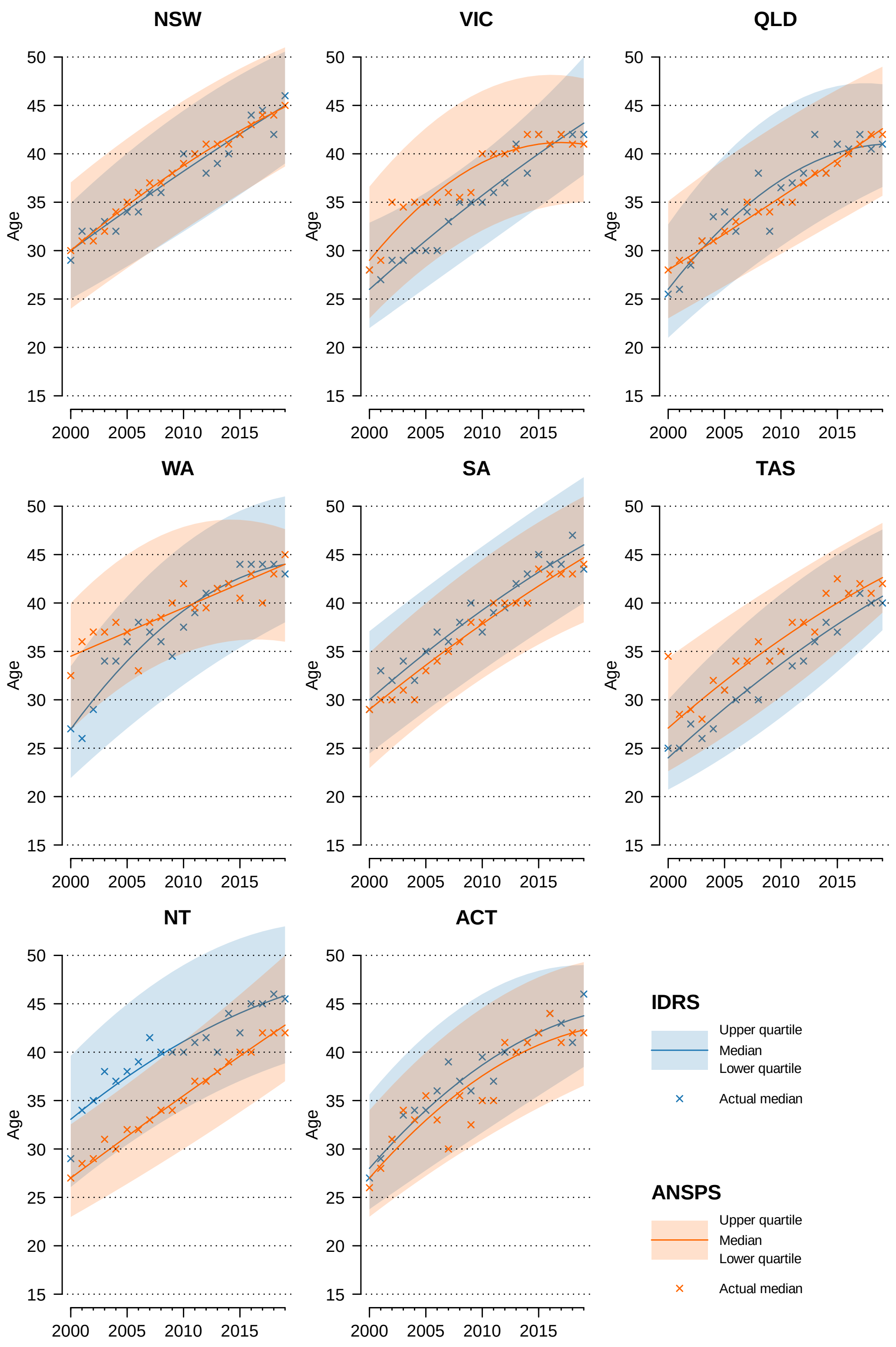

E2. Age of injecting initiation among IDRS and ANSPS participants.

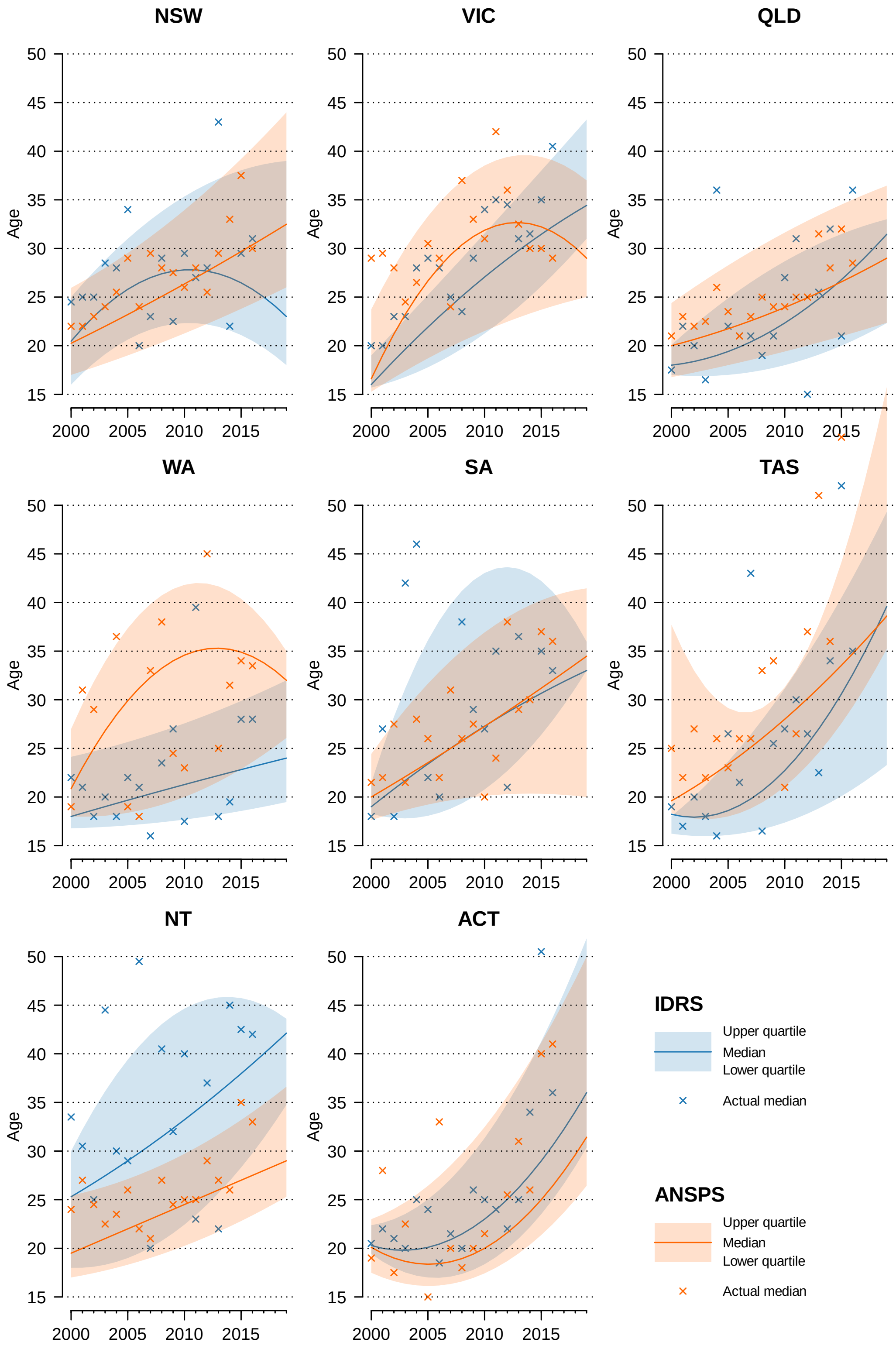

E3. Time since injecting drug use initiation among IDRS and ANSPS participants.

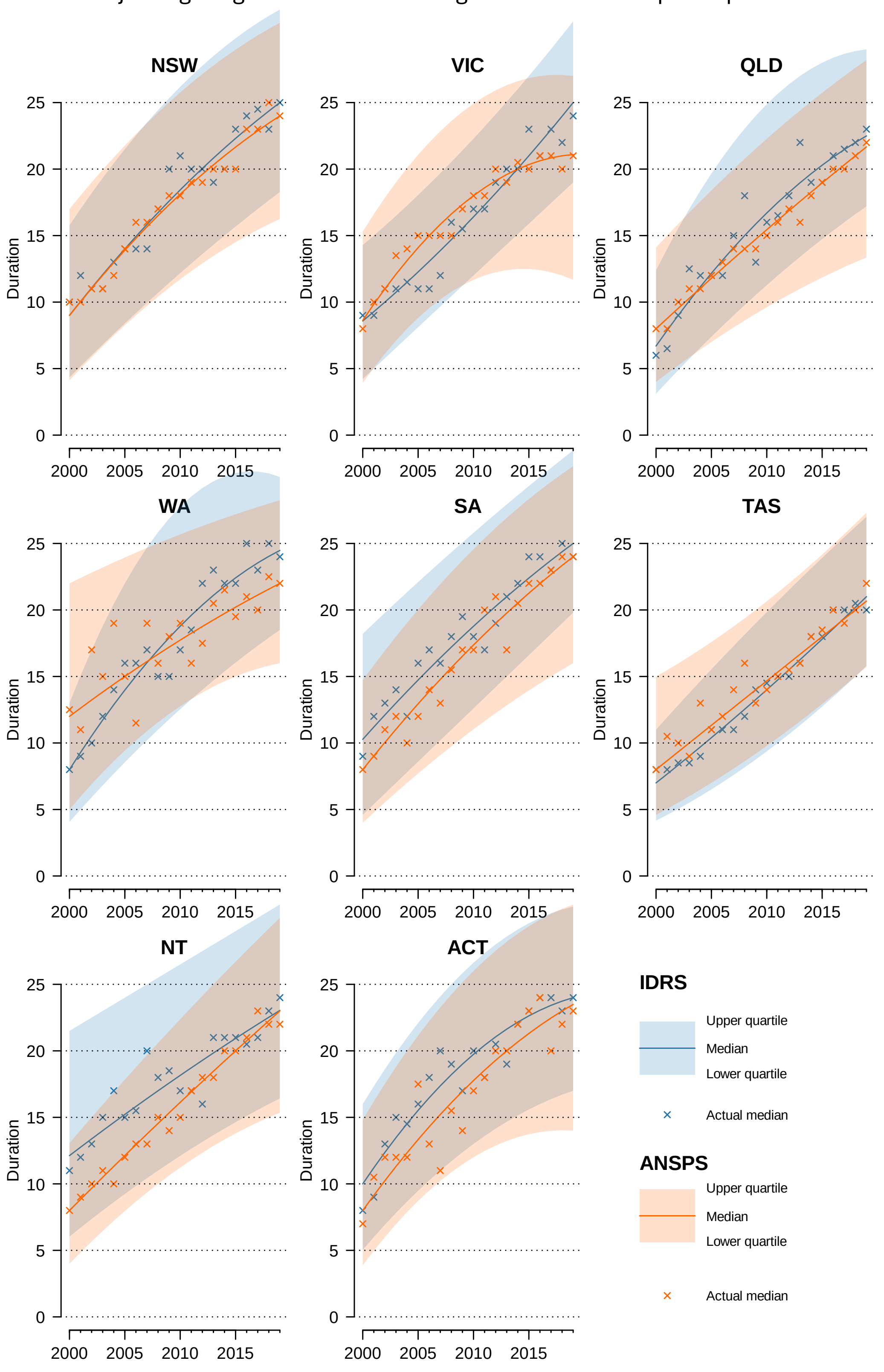

Appendix F. Age of initiation among IDRS and ANSPS participants, without restricting the sample to those who initiated injecting drug use within 3 years of the survey.

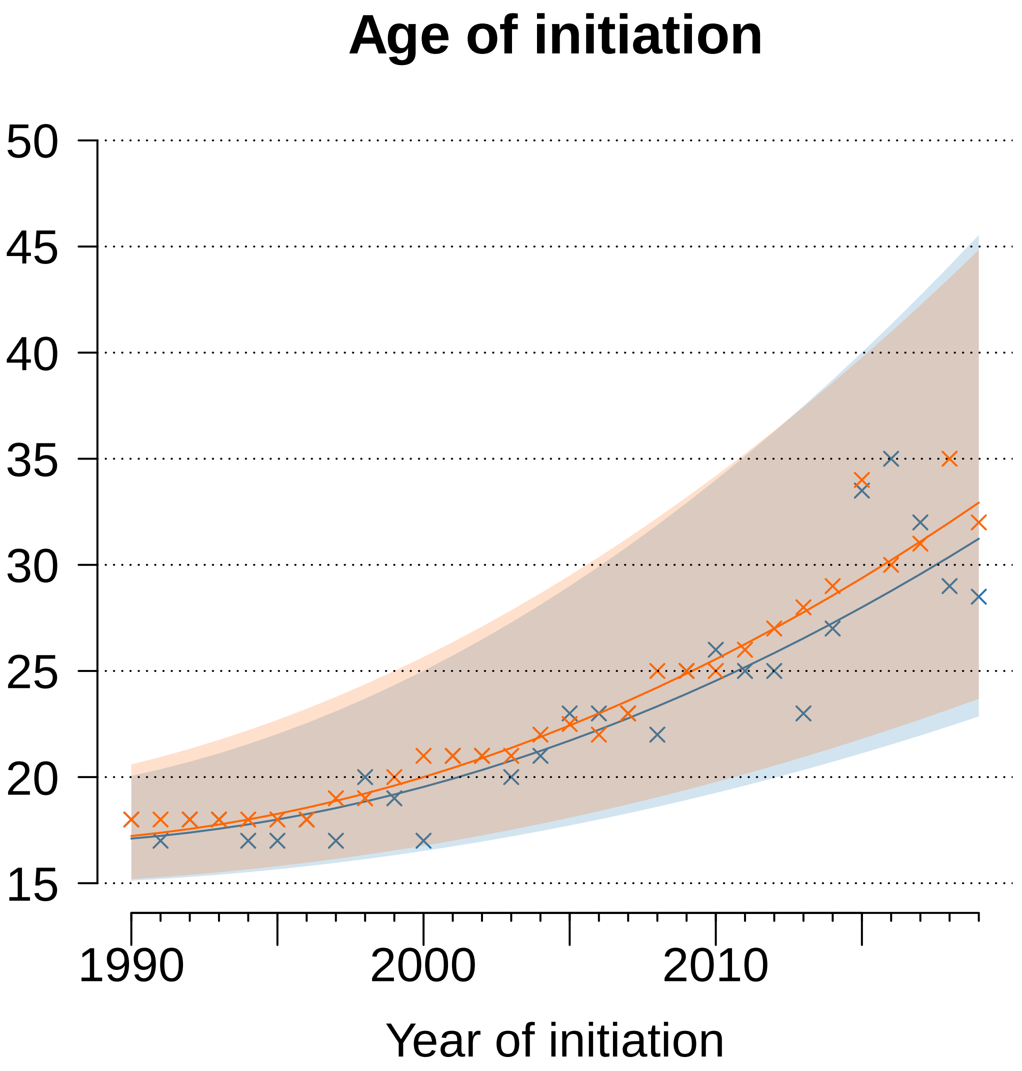

Appendix G. Model information.

G1. Example calculations of pairwise ratios of new initiators.

Survey year sy	Initiation year 1 y1	Initiation year 2 y2	Difference in time d=y2-y1	Proportion of initiates from y1 remaining in population in y2 $p=e^{(-d/\lambda)}$	Number starting injecting in y1 N1	Number starting injecting in y2 N2	Adjusted number in y1 aN1=N1/p	Ratio R=N2/aN1
2000	1990	1991	1	0.94	50	60	53.4	1.12
2000	1990	1992	2	0.88	70	85	80.0	1.06
2000	1991	1993	3	0.82	80	90	97.7	0.92

G2. Heatmap of the pairwise ratios of number of people beginning to inject drugs in Australia, among IDRS (top) and ANSPS (bottom) participants.

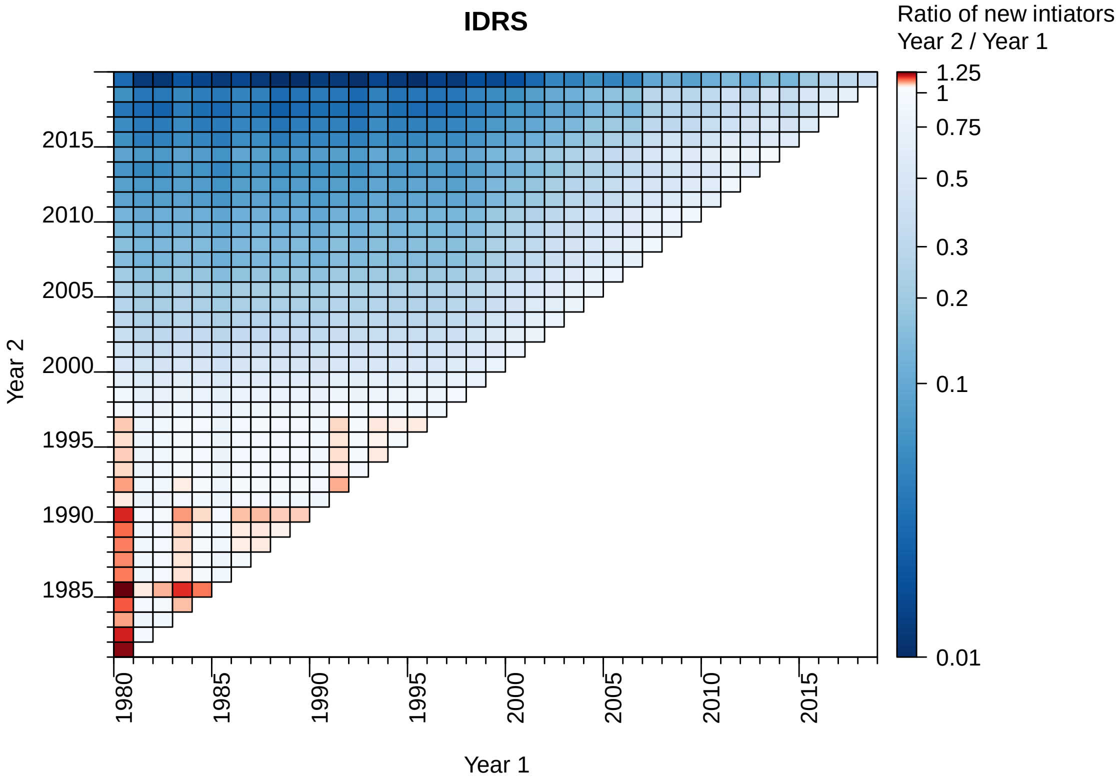

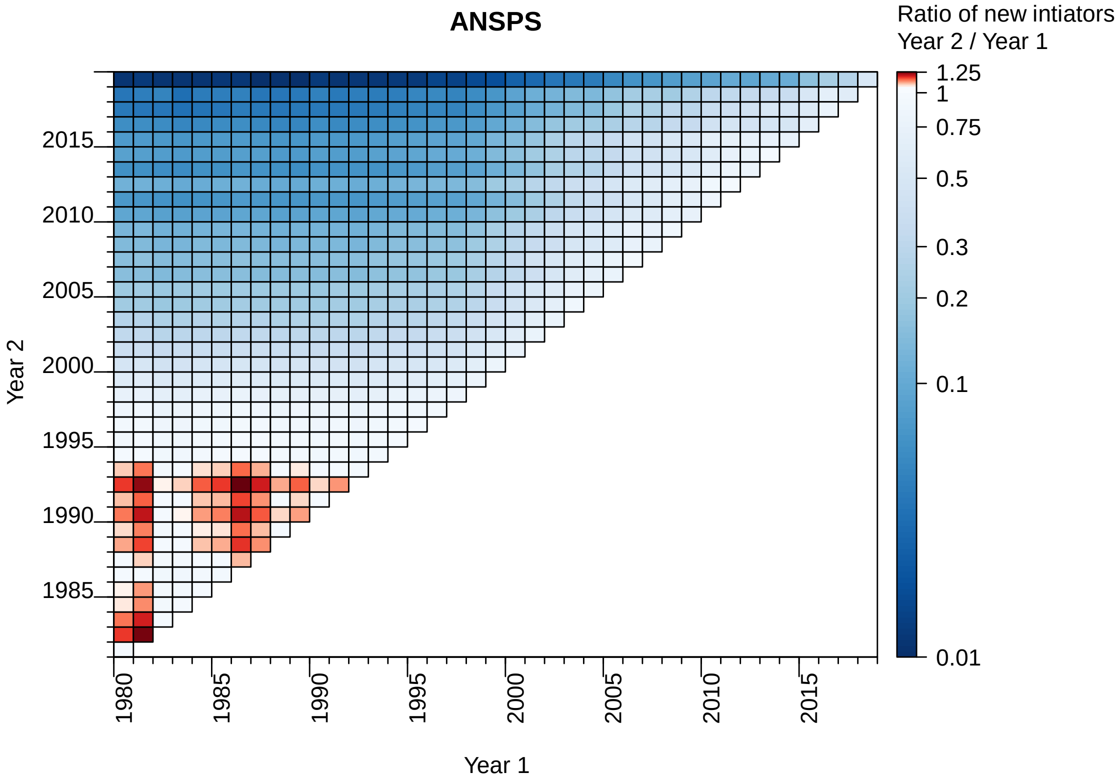

G3. Estimate of the ratio of people injecting drugs for the first time in Australia, compared to 1980. The band represents the 95% prediction interval.

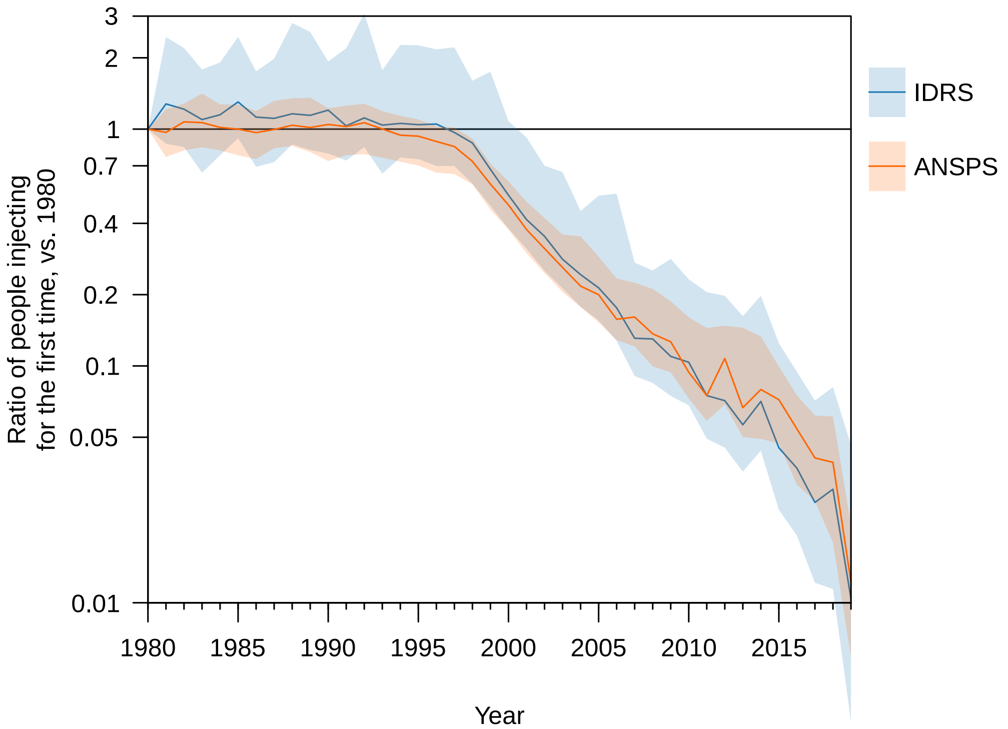

G4. Sensitivity analysis of the effect of altering the assumed mean duration of injecting drug use on the ratio of people injecting drugs for the first time in Australia, using IDRS (top) and ANSPS (bottom) data.

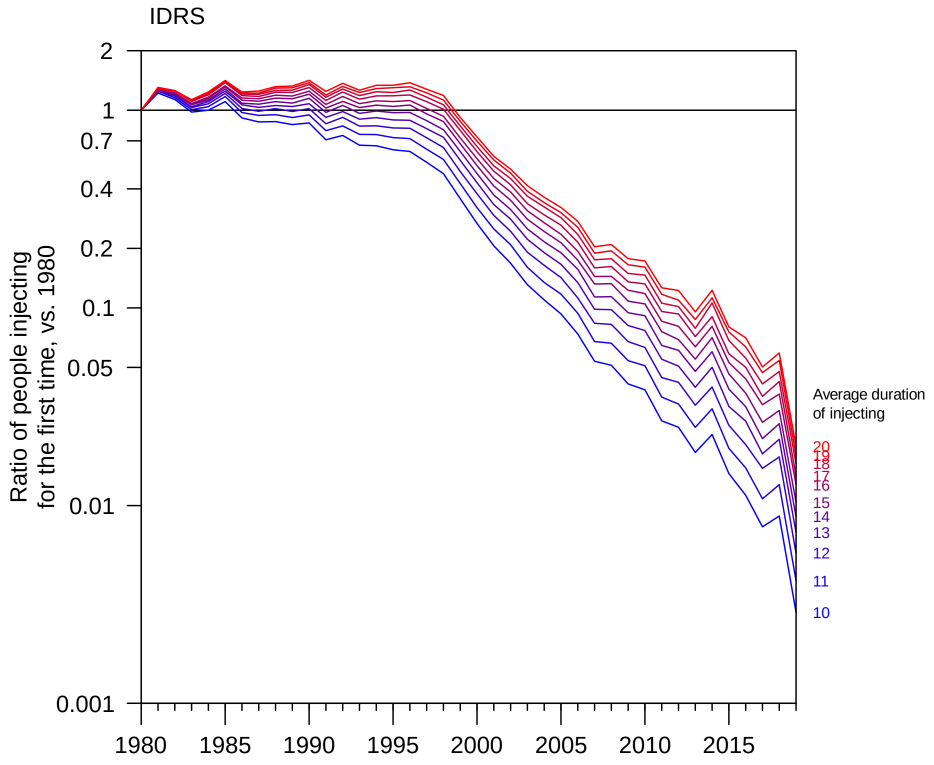

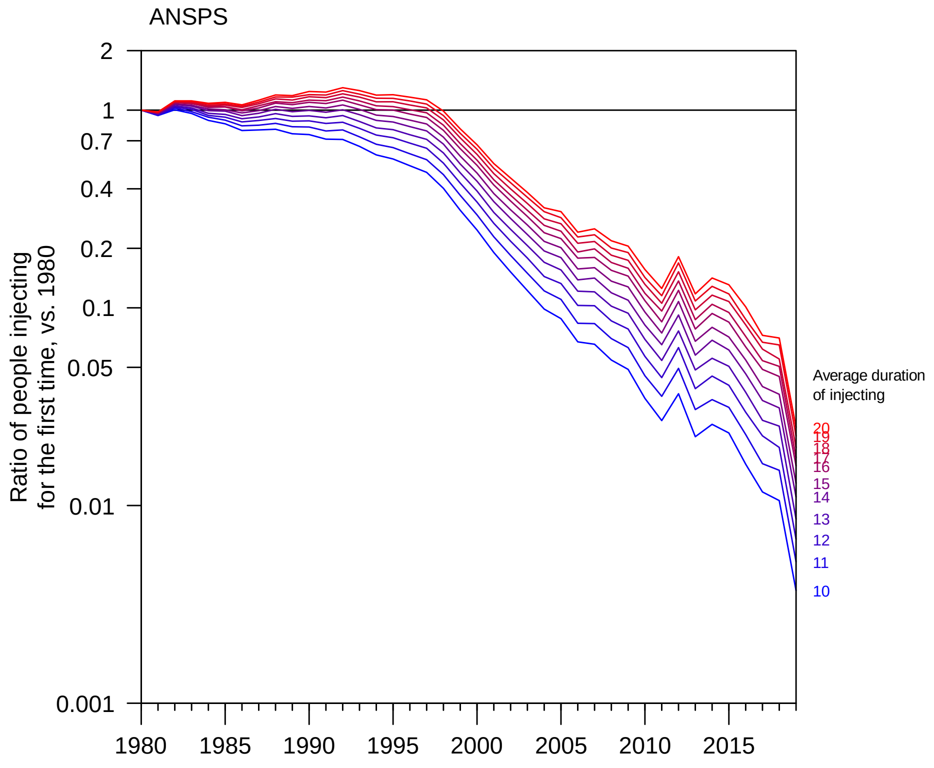

G5. Sensitivity analysis restricting the survey years to model the number of people injecting drugs for the first time, using IDRS data.

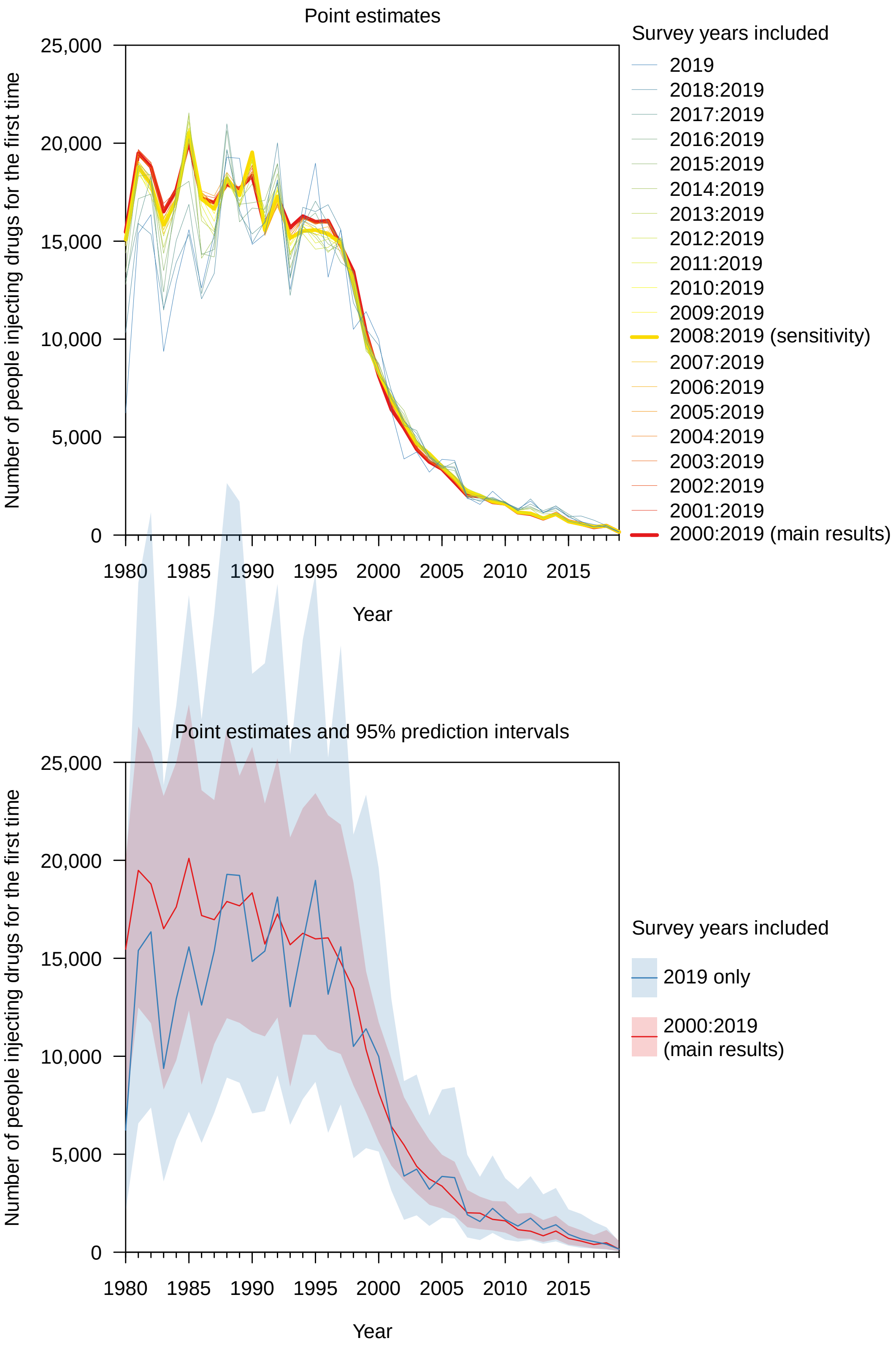

G6. Sensitivity analysis restricting the survey years to model the number of people injecting drugs for the first time, using ANSPS data.

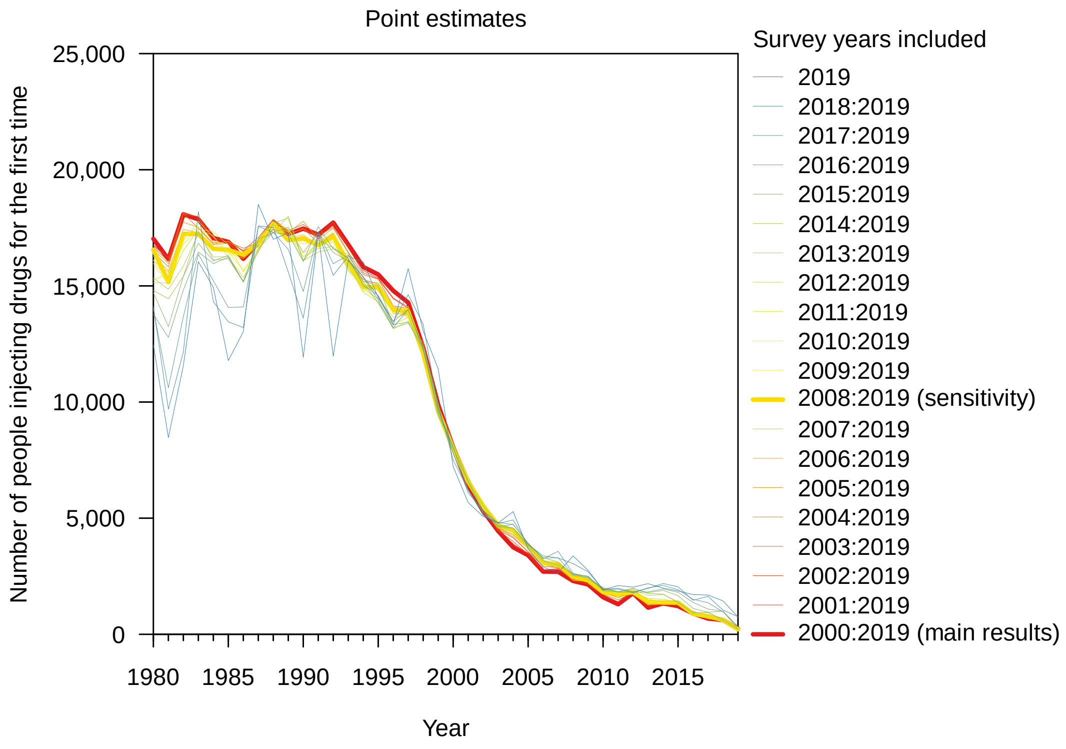

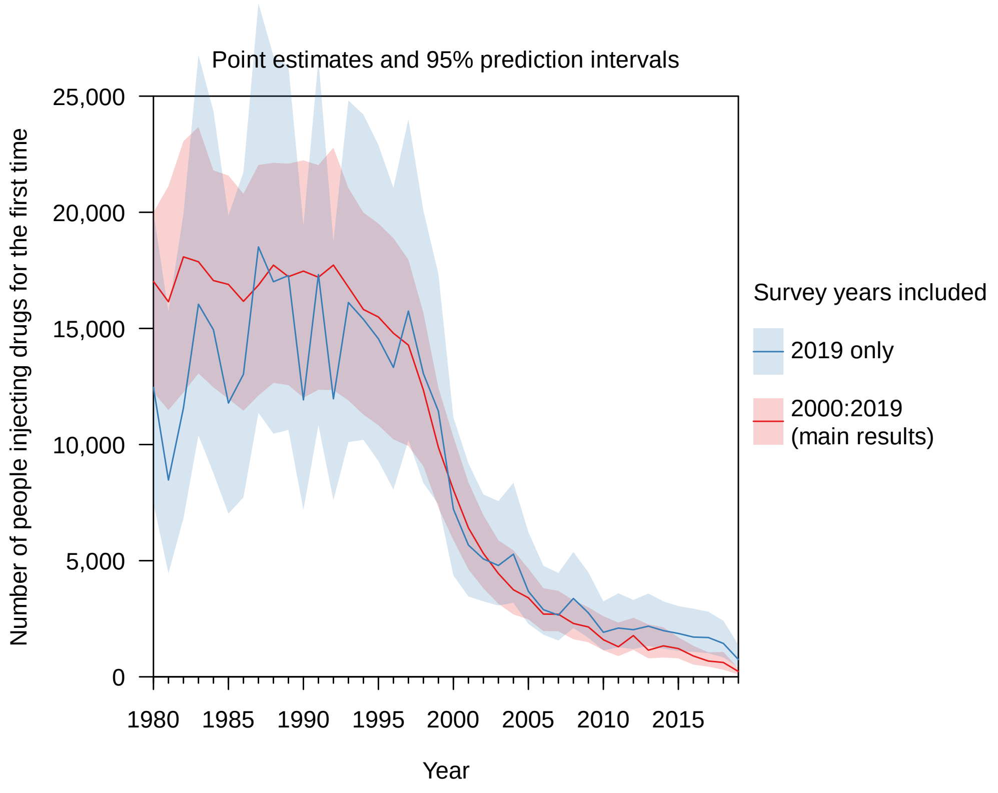

Appendix H. The modelled number of people injecting drugs for the first time in Australia.

H1. Using IDRS data.

Year	Australia	NSW	Victoria	Queensland	WA
**1980**	15570 (7240-20000)	8170 (3410-11490)	2790 (1420-4280)	2870 (1720-4230)	1110 (670-2050)
**1981**	19890 (12490-26830)	8610 (4940-12780)	3250 (1300-4810)	2050 (760-3110)	1160 (560-1840)
**1982**	18900 (11680-25550)	8810 (4910-12890)	4540 (2660-7310)	2610 (1320-3960)	1260 (560-1940)
**1983**	17080 (8300-23280)	8200 (5170-12160)	4330 (2550-7150)	2530 (1120-3790)	1320 (780-2170)
**1984**	17900 (9790-25010)	8390 (5770-12850)	3680 (1470-5340)	2330 (1440-3780)	1630 (650-2590)
**1985**	20280 (12330-27960)	8170 (5320-12130)	4690 (1990-6860)	1990 (1060-2990)	1520 (970-2400)
**1986**	17500 (8550-23580)	6910 (3930-10050)	3860 (2250-5860)	2370 (1460-3650)	1630 (730-2480)
**1987**	17290 (10630-23070)	7300 (4870-10790)	4400 (2820-6950)	2550 (1470-3710)	1330 (730-2000)
**1988**	18070 (11950-26750)	7220 (5110-11790)	4210 (2170-6080)	2830 (1910-4350)	1650 (610-2360)
**1989**	17800 (11700-24310)	6120 (4190-9460)	4150 (1990-6200)	2950 (1930-4260)	1780 (1040-2540)
**1990**	18730 (11240-25790)	6890 (4130-9710)	3760 (1900-5460)	2930 (1760-4180)	1810 (880-2740)
**1991**	16070 (11010-22900)	6610 (4620-10610)	4190 (2100-5880)	3150 (1980-4520)	1550 (780-2370)
**1992**	17370 (11980-25210)	6860 (4460-9480)	4360 (2100-6270)	3220 (2000-4620)	1690 (1000-2450)
**1993**	16190 (8450-21170)	6270 (4050-9220)	3860 (2310-5740)	3120 (1930-4380)	1500 (790-2190)
**1994**	16460 (11110-22660)	6080 (3800-8280)	4090 (2490-6040)	2990 (1890-4140)	1270 (770-1960)
**1995**	16250 (11090-23430)	5780 (3670-8220)	4390 (2310-6090)	2770 (1780-3880)	1440 (890-2190)
**1996**	16360 (10360-22300)	5560 (3440-7820)	3950 (2380-5640)	2610 (1580-3660)	1370 (720-1980)
**1997**	15060 (10110-21820)	5280 (3640-7850)	3720 (2230-5560)	2630 (1540-3710)	1220 (800-1780)
**1998**	13610 (8510-18880)	4510 (2860-6320)	3110 (1830-4770)	2360 (1530-3330)	1120 (700-1640)
**1999**	10520 (7130-14340)	3540 (2320-5060)	2350 (1290-3530)	2030 (1330-3050)	840 (520-1280)
**2000**	8190 (5630-11740)	2960 (1720-4050)	2020 (1260-3110)	1490 (980-2170)	660 (430-1100)
**2001**	6460 (4420-9830)	2480 (1650-3770)	1440 (720-2120)	1220 (700-1800)	580 (340-880)
**2002**	5490 (3650-7910)	1750 (1140-2760)	1090 (630-1740)	1080 (710-1590)	490 (300-790)
**2003**	4390 (3000-6750)	1570 (860-2400)	1100 (670-1940)	820 (500-1300)	390 (220-590)
**2004**	3780 (2420-5740)	1260 (830-2420)	990 (590-1650)	670 (410-1140)	300 (190-590)
**2005**	3330 (2230-4980)	1160 (710-1920)	880 (560-1740)	670 (420-1060)	310 (180-510)
**2006**	2740 (1870-4620)	770 (460-1480)	660 (400-1170)	570 (330-890)	310 (180-530)
**2007**	2040 (1270-3180)	890 (530-1520)	510 (290-970)	560 (300-890)	310 (190-520)
**2008**	2020 (1180-2830)	930 (520-1410)	490 (280-900)	400 (240-700)	260 (160-430)
**2009**	1710 (1110-2610)	840 (450-1390)	470 (260-850)	380 (240-700)	210 (120-370)
**2010**	1610 (1000-2590)	580 (360-1150)	270 (110-440)	390 (230-620)	160 (90-280)
**2011**	1170 (700-1970)	450 (260-860)	310 (180-730)	260 (160-490)	210 (110-390)
**2012**	1110 (680-2010)	590 (290-1030)	330 (150-570)	430 (240-690)	210 (110-360)
**2013**	880 (520-1640)	460 (260-1020)	210 (120-440)	330 (170-590)	120 (70-260)
**2014**	1100 (660-1860)	640 (300-1190)	280 (150-620)	260 (140-630)	190 (90-390)
**2015**	700 (380-1350)	640 (190-1320)	100 (40-240)	280 (140-560)	230 (130-420)
**2016**	580 (300-1120)	490 (200-930)	170 (90-380)	280 (130-580)	130 (70-300)
**2017**	410 (190-880)	250 (110-600)	170 (60-370)	360 (150-750)	110 (50-230)
**2018**	470 (160-1130)	160 (60-350)	170 (40-540)	180 (60-450)	100 (40-320)
**2019**	160 (50-580)	140 (50-530)	220 (40-1090)	70 (30-190)	60 (10-320)

Year	SA	Tasmania	ACT	NT
**1980**	1220 (680-2040)	150 (70-300)	310 (120-520)	120 (40-230)
**1981**	1200 (670-1940)	240 (100-460)	260 (120-470)	80 (30-160)
**1982**	1360 (590-2110)	150 (70-260)	360 (140-720)	110 (40-250)
**1983**	1630 (740-2760)	170 (90-400)	360 (150-620)	100 (40-250)
**1984**	1170 (510-1870)	180 (90-390)	430 (220-830)	80 (30-170)
**1985**	1170 (430-1730)	150 (60-270)	280 (110-530)	80 (40-160)
**1986**	1320 (790-2150)	140 (60-250)	230 (110-420)	110 (40-220)
**1987**	1290 (810-2320)	120 (60-200)	270 (150-600)	80 (40-150)
**1988**	1430 (890-2550)	150 (80-360)	230 (90-410)	60 (30-110)
**1989**	1520 (850-2310)	180 (90-370)	270 (110-490)	80 (30-140)
**1990**	1380 (510-2000)	190 (60-320)	220 (70-350)	100 (50-210)
**1991**	1190 (690-1940)	180 (70-380)	260 (60-470)	80 (50-190)
**1992**	1210 (490-1790)	150 (50-260)	250 (130-470)	70 (30-130)
**1993**	1180 (710-1790)	200 (80-390)	310 (150-520)	70 (40-140)
**1994**	1020 (560-1550)	140 (50-260)	160 (90-350)	60 (20-120)
**1995**	940 (500-1420)	180 (60-340)	250 (100-390)	60 (20-130)
**1996**	1040 (420-1480)	150 (50-230)	170 (70-290)	70 (30-150)
**1997**	970 (580-1490)	130 (40-210)	200 (90-340)	70 (30-130)
**1998**	760 (450-1450)	150 (60-260)	160 (80-280)	40 (20-80)
**1999**	610 (260-890)	90 (40-190)	160 (90-320)	60 (30-120)
**2000**	610 (200-890)	100 (50-180)	90 (40-160)	30 (10-60)
**2001**	420 (230-710)	90 (40-150)	90 (40-170)	30 (10-70)
**2002**	360 (210-580)	70 (40-170)	80 (30-140)	20 (10-40)
**2003**	320 (190-620)	50 (20-110)	80 (40-140)	20 (10-60)
**2004**	270 (110-530)	50 (20-130)	90 (40-170)	20 (10-50)
**2005**	180 (90-310)	50 (30-120)	50 (20-100)	20 (10-30)
**2006**	220 (70-340)	40 (10-80)	60 (30-130)	20 (10-30)
**2007**	170 (100-320)	30 (10-60)	60 (30-130)	10 (0-30)
**2008**	140 (70-260)	40 (20-80)	40 (20-90)	20 (0-30)
**2009**	160 (80-290)	30 (10-60)	60 (30-120)	10 (10-30)
**2010**	100 (50-250)	30 (10-70)	30 (10-70)	10 (0-20)
**2011**	90 (40-180)	30 (10-70)	30 (10-60)	10 (0-50)
**2012**	130 (60-250)	20 (10-50)	20 (10-50)	10 (0-40)
**2013**	100 (20-190)	10 (0-60)	20 (10-60)	10 (0-40)
**2014**	90 (40-190)	20 (10-60)	30 (10-80)	20 (10-70)
**2015**	140 (60-310)	20 (0-70)	20 (10-50)	10 (0-20)
**2016**	70 (30-170)	NA (NA-NA)	20 (10-80)	10 (0-30)
**2017**	50 (20-110)	10 (0-60)	30 (10-120)	10 (0-20)
**2018**	70 (10-400)	NA (NA-NA)	20 (0-50)	NA (NA-NA)
**2019**	20 (0-100)	NA (NA-NA)	10 (0-60)	NA (NA-NA)

H2. Using ANSPS data.

Year	Australia	NSW	Victoria	Queensland	WA
**1980**	17020 (12250-20000)	7310 (2950-12400)	2700 (970-4760)	2550 (1300-4310)	1490 (520-2580)
**1981**	16480 (11480-21130)	9490 (3420-15040)	3150 (1670-8000)	2410 (1040-3960)	1400 (540-3330)
**1982**	18260 (12240-23060)	8510 (4780-19950)	3720 (1610-6720)	2580 (1130-4560)	2160 (850-3890)
**1983**	18130 (13060-23670)	8070 (2750-13420)	4440 (1340-7460)	1890 (960-2960)	1220 (430-3260)
**1984**	17300 (12460-21800)	6430 (2440-10380)	4060 (1730-7220)	2640 (1150-4220)	1340 (350-3470)
**1985**	17010 (11970-21580)	7620 (1900-11810)	4460 (1070-7520)	3090 (1690-5740)	1560 (790-7060)
**1986**	16450 (11460-20800)	6630 (1600-11650)	4290 (1790-6930)	2760 (1470-4670)	1860 (580-3230)
**1987**	16950 (12110-22040)	7160 (3130-12020)	5060 (2440-8040)	2250 (1030-3720)	1170 (270-2070)
**1988**	17670 (12650-22130)	7650 (3710-13270)	5170 (2580-8090)	2540 (1350-8230)	1220 (610-2460)
**1989**	17290 (12560-22090)	7430 (4060-13020)	5240 (2270-8920)	2100 (750-3480)	1560 (730-3180)
**1990**	17810 (12020-22240)	6430 (3530-12190)	4760 (2250-7520)	3220 (1210-5440)	1590 (580-2620)
**1991**	17450 (12360-22030)	6390 (3200-10390)	3920 (1700-6220)	3580 (2090-5940)	1400 (550-2470)
**1992**	18110 (12340-22780)	6440 (3860-11660)	4020 (1990-6550)	2720 (1430-4610)	1860 (960-4800)
**1993**	17040 (11900-21040)	4530 (2070-7710)	3880 (2110-7170)	3250 (1590-5680)	1590 (490-2630)
**1994**	16040 (11290-19990)	6660 (3560-11770)	4130 (1880-6280)	2680 (1280-4520)	1180 (590-2420)
**1995**	15890 (10830-19510)	5500 (2280-8680)	4340 (1200-6410)	2870 (1620-4630)	1450 (710-2800)
**1996**	15090 (10220-18890)	6030 (2350-9340)	3840 (1900-6680)	3240 (1960-5280)	1210 (400-2060)
**1997**	14370 (9940-17960)	5950 (2860-9710)	4380 (2280-7060)	2550 (1300-4050)	1110 (580-2310)
**1998**	12440 (9070-15670)	5890 (3460-11150)	3300 (910-5070)	2700 (1550-4530)	1200 (450-1970)
**1999**	9970 (7280-12450)	3180 (950-4860)	2540 (1320-4150)	2510 (1190-4220)	820 (220-1430)
**2000**	8140 (5890-10330)	3460 (1190-5510)	1630 (960-4540)	1450 (790-2370)	490 (130-840)
**2001**	6410 (4620-8380)	2110 (980-3580)	1130 (430-2090)	1020 (590-2060)	460 (160-850)
**2002**	5330 (3810-6960)	2500 (1100-4180)	1250 (610-2390)	930 (520-1750)	520 (220-1610)
**2003**	4430 (3140-5880)	1310 (710-3390)	850 (430-1560)	630 (350-1340)	320 (110-550)
**2004**	3700 (2690-5450)	930 (420-1590)	630 (190-1000)	610 (340-1560)	410 (140-1280)
**2005**	3400 (2470-4660)	1060 (500-3150)	660 (320-1440)	430 (210-720)	360 (130-790)
**2006**	2680 (1970-3820)	900 (510-2410)	520 (260-990)	430 (220-750)	300 (100-590)
**2007**	2740 (1960-3700)	810 (230-1410)	380 (190-750)	460 (210-810)	240 (60-590)
**2008**	2330 (1620-3300)	700 (270-1290)	440 (230-1150)	460 (180-990)	340 (130-990)
**2009**	2150 (1490-3000)	600 (260-1080)	390 (190-1020)	350 (130-660)	210 (70-460)
**2010**	1600 (1140-2610)	890 (410-2020)	330 (160-870)	450 (150-1170)	230 (70-620)
**2011**	1280 (890-2340)	480 (220-1390)	240 (90-510)	350 (110-740)	230 (70-600)
**2012**	1830 (1160-2540)	760 (290-1660)	230 (100-490)	270 (90-650)	250 (60-730)
**2013**	1130 (790-2260)	560 (180-1260)	310 (130-750)	190 (60-420)	150 (40-350)
**2014**	1350 (830-2130)	460 (130-1070)	250 (100-820)	530 (140-1720)	170 (50-400)
**2015**	1230 (790-1700)	470 (80-1680)	150 (50-330)	180 (50-520)	210 (40-700)
**2016**	920 (520-1340)	430 (90-1850)	150 (30-480)	310 (50-1680)	150 (30-510)
**2017**	700 (440-1060)	460 (100-2160)	310 (50-1680)	140 (40-390)	250 (50-870)
**2018**	670 (310-1070)	230 (50-770)	NA (NA-NA)	NA (NA-NA)	200 (40-690)
**2019**	210 (100-370)	NA (NA-NA)	120 (20-770)	NA (NA-NA)	90 (10-370)

Year	SA	Tasmania	ACT	NT
**1980**	1540 (560-3190)	120 (50-240)	300 (160-510)	110 (40-210)
**1981**	1750 (460-2810)	130 (70-220)	360 (190-550)	150 (70-300)
**1982**	1730 (550-2860)	120 (60-280)	360 (170-550)	100 (40-160)
**1983**	1520 (490-2630)	120 (60-210)	320 (160-530)	90 (40-160)
**1984**	1270 (570-2320)	180 (100-290)	310 (150-450)	80 (40-150)
**1985**	1130 (400-2310)	100 (50-190)	650 (370-990)	90 (40-180)
**1986**	1280 (320-2110)	120 (60-200)	220 (120-350)	100 (40-160)
**1987**	1160 (470-2640)	100 (60-190)	250 (110-360)	110 (30-190)
**1988**	1420 (540-2680)	120 (40-190)	310 (170-490)	100 (50-180)
**1989**	1420 (720-2720)	120 (60-180)	320 (160-470)	80 (40-140)
**1990**	1210 (220-2050)	190 (120-320)	310 (190-500)	70 (30-130)
**1991**	1080 (610-2580)	140 (80-220)	210 (110-350)	70 (30-130)
**1992**	1010 (190-1750)	170 (80-250)	260 (160-530)	80 (40-150)
**1993**	1280 (490-2100)	190 (80-280)	230 (130-380)	60 (10-110)
**1994**	1110 (440-2020)	170 (90-270)	320 (160-480)	80 (30-140)
**1995**	1090 (550-2610)	170 (90-250)	270 (140-420)	70 (30-140)
**1996**	1120 (620-2100)	210 (110-290)	280 (120-400)	80 (30-140)
**1997**	860 (400-1590)	200 (110-280)	140 (70-200)	70 (30-130)
**1998**	850 (380-1520)	160 (80-220)	150 (90-260)	60 (30-120)
**1999**	520 (170-960)	110 (60-160)	120 (70-210)	60 (40-120)
**2000**	500 (170-1000)	90 (50-180)	90 (50-200)	40 (20-90)
**2001**	350 (140-680)	100 (60-160)	90 (50-190)	30 (10-60)
**2002**	370 (150-750)	70 (40-120)	70 (30-110)	30 (20-60)
**2003**	340 (190-850)	90 (50-140)	70 (30-110)	20 (10-40)
**2004**	190 (80-350)	60 (30-110)	80 (40-130)	20 (10-30)
**2005**	230 (90-430)	50 (30-90)	50 (30-90)	20 (10-40)
**2006**	280 (120-630)	30 (20-60)	40 (20-80)	10 (10-40)
**2007**	170 (70-380)	30 (20-60)	40 (20-70)	20 (10-40)
**2008**	160 (60-350)	20 (10-50)	30 (20-70)	20 (10-40)
**2009**	110 (50-240)	20 (10-40)	40 (20-70)	10 (10-20)
**2010**	120 (50-290)	30 (10-60)	30 (20-70)	10 (0-30)
**2011**	110 (40-230)	20 (10-30)	20 (10-60)	10 (0-20)
**2012**	120 (40-270)	20 (10-40)	20 (10-50)	10 (0-30)
**2013**	90 (40-210)	20 (10-50)	20 (10-50)	10 (0-30)
**2014**	70 (10-320)	20 (0-110)	20 (10-60)	10 (0-30)
**2015**	170 (40-580)	10 (0-50)	40 (10-100)	10 (0-30)
**2016**	70 (10-350)	10 (0-30)	20 (10-70)	20 (0-110)
**2017**	60 (10-370)	NA (NA-NA)	NA (NA-NA)	10 (0-30)
**2018**	100 (20-600)	NA (NA-NA)	NA (NA-NA)	10 (0-70)
**2019**	NA (NA-NA)	NA (NA-NA)	NA (NA-NA)	NA (NA-NA)

Appendix I. Modelled number of men and women injecting drugs for the first time in Australia.

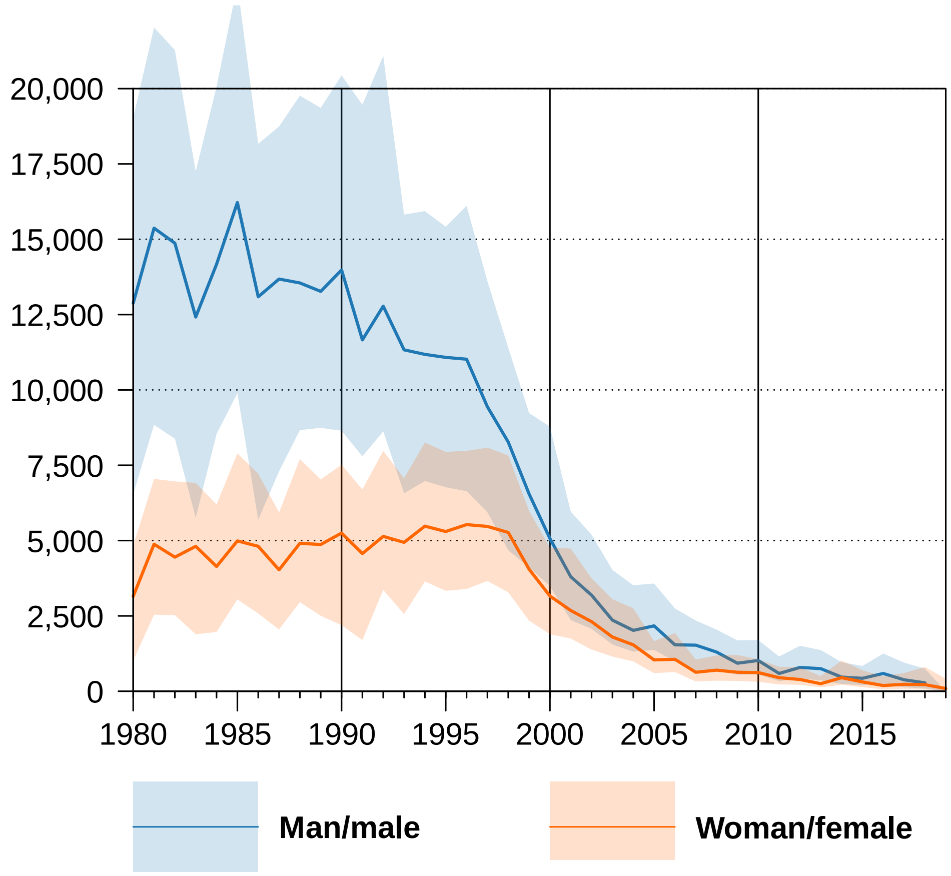

Appendix J. Modelled number of people initiating injecting drug use in each Australian jurisdiction.

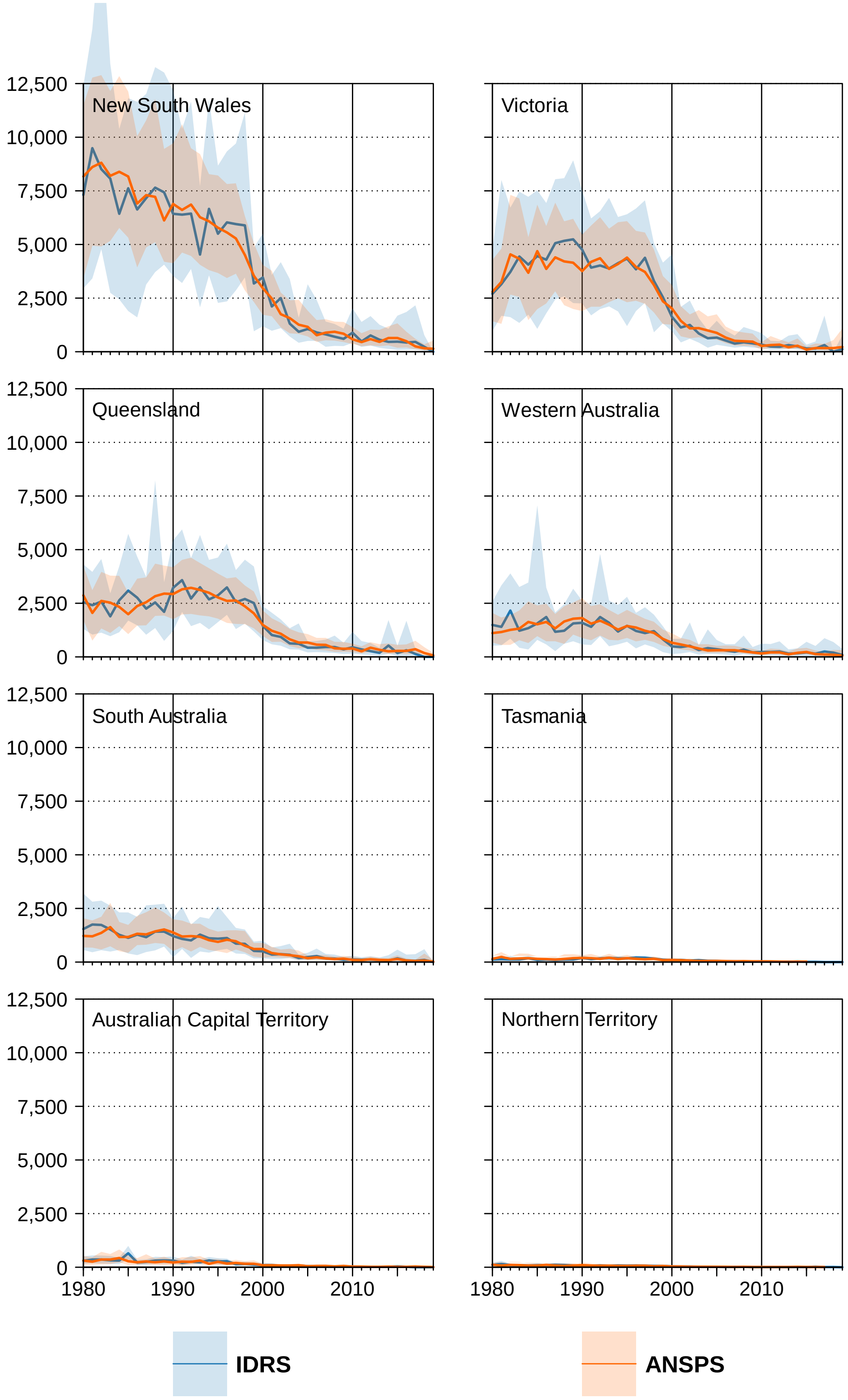

Supplement: Price Changes in age etc Supplementary revised [file mmc1.pdf]
